# TFIID dependency of steady-state mRNA transcription altered epigenetically by simultaneous functional loss of Taf1 and Spt3 is Hsp104-dependent

**DOI:** 10.1371/journal.pone.0281233

**Published:** 2023-02-09

**Authors:** Ryo Iwami, Naoki Takai, Minenosuke Matsutani, Yuh Shiwa, Haruki Kokubo, Koji Kasahara, Tetsuro Kokubo

**Affiliations:** 1 Molecular and Cellular Biology Laboratory, Graduate School of Medical Life Science, Yokohama City University, Yokohama, Kanagawa, Japan; 2 NODAI Genome Research Center, Tokyo University of Agriculture, Setagaya-ku, Tokyo, Japan; 3 Department of Molecular Microbiology, Faculty of Life Sciences, Tokyo University of Agriculture, Setagaya-ku, Tokyo, Japan; 4 Faculty of Medicine, The University of Tokyo, Bunkyo-ku, Tokyo, Japan; Southern Illinois University School of Medicine, UNITED STATES

## Abstract

In *Saccharomyces cerevisiae*, class II gene promoters have been divided into two subclasses, TFIID- and SAGA-dominated promoters or TFIID-dependent and coactivator-redundant promoters, depending on the experimental methods used to measure mRNA levels. A prior study demonstrated that Spt3, a TBP-delivering subunit of SAGA, functionally regulates the *PGK1* promoter via two mechanisms: by stimulating TATA box-dependent transcriptional activity and conferring Taf1/TFIID independence. However, only the former could be restored by plasmid-borne *SPT3*. In the present study, we sought to determine why ectopically expressed *SPT3* is unable to restore Taf1/TFIID independence to the *PGK1* promoter, identifying that this function was dependent on the construction protocol for the *SPT3 taf1* strain. Specifically, simultaneous functional loss of Spt3 and Taf1 during strain construction was a prerequisite to render the *PGK1* promoter Taf1/TFIID-dependent in this strain. Intriguingly, genetic approaches revealed that an as-yet unidentified trans-acting factor reprogrammed the transcriptional mode of the *PGK1* promoter from the Taf1/TFIID-independent state to the Taf1/TFIID-dependent state. This factor was generated in the haploid *SPT3 taf1* strain in an Hsp104-dependent manner and inherited meiotically in a non-Mendelian fashion. Furthermore, RNA-seq analyses demonstrated that this factor likely affects the transcription mode of not only the *PGK1* promoter, but also of many other class II gene promoters. Collectively, these findings suggest that a prion or biomolecular condensate is generated in a Hsp104-dependent manner upon simultaneous functional loss of TFIID and SAGA, and could alter the roles of these transcription complexes on a wide variety of class II gene promoters without altering their primary sequences. Therefore, these findings could provide the first evidence that TFIID dependence of class II gene transcription can be altered epigenetically, at least in *Saccharomyces cerevisiae*.

## Introduction

To initiate transcription in eukaryotes, TATA-binding protein (TBP) first binds to the TATA box or TATA-like element [1], and subsequently recruits other general transcription factors, Mediator and RNA polymerase II, to assemble the preinitiation complex (PIC) at the core promoter region [2–5]. TBP is an integral subunit of TFIID and is generally delivered to the promoter of class II genes via TFIID or SAGA, which are related multi-protein complexes containing five Tafs as common components [3, 6–8].

Class II genes can be divided into two subclasses, TFIID- or SAGA-dominated genes, depending on whether steady-state mRNA levels are impaired more robustly by TFIID or SAGA mutations, respectively [9]. A recent study potentially challenged this dogma, demonstrating that nascent mRNA levels of most (~87%) analyzable class II genes are significantly affected by depletion of TFIID, but not of SAGA, while the remainder of class II genes (~13%) are affected modestly by single depletion of either TFIID or SAGA, but significantly affected by depletion of both TFIID and SAGA [10]. These newly defined types of class II genes are designated as “TFIID-dependent” and “coactivator-redundant (CR),” respectively [10]. TBP is delivered to the TATA element of TFIID-dependent genes solely via TFIID, while either TFIID or SAGA facilitates delivery of TBP to CR genes [10]. Notably, TFIID-dependent and CR genes overlap with, but are not identical to, TFIID- and SAGA-dominated genes, respectively. This seeming discrepancy could be attributed to the experimental methods exploited in these two studies [9, 10], which either measured nascent [10] or steady-state [9] mRNA levels for classification. Although it is certain that at least two distinct types of class II gene promoters exist, the distinct roles of TFIID and SAGA in transcription from class II promoters remain incompletely understood.

To facilitate transcription, activators that bind DNA at a distance from their target promoters make physical contact with the general transcription machinery assembled near the transcription start site by looping out the intervening DNA [11, 12]. During this event, the entire transcriptional machinery, including activators, coactivators, RNA polymerase II, and even the transcribed RNA, could form large separated assemblies in a liquid-like state, referred to as transcriptional condensates, primarily via weak multivalent interactions among intrinsically disordered regions (IDRs) of these components [13–17]. These condensates enable efficient transcription by increasing localized concentrations of transcription factors and DNA. In living cells, activators bind to only a limited number of the genomic sites that contain their cognate DNA recognition motifs. In this context, it is notable that the long IDRs of the two distinct types of yeast activators, Msn2 and Yap1, play a crucial role in achieving this selective binding *in vivo* [18]. Thus, transcriptional condensates could also provide a means for activators to efficiently locate their target sites, for example by enabling a facilitated diffusion search within a restricted zone of the nucleus [19]. Furthermore, a recent study revealed that Taf14, a histone reader YEATS family protein that physically associates with not only TFIID but also with several other transcription complexes, undergoes liquid-liquid phase separation *in vitro* [20]. This suggests that Taf14 could serve as a central hub in formation of some transcriptional condensates. Despite these recent findings, it is currently unknown whether the two types of class II gene promoters regulated differentially by TFIID and SAGA can form distinct promoter type-specific transcriptional condensates that are differentially dependent on TFIID and/or SAGA in transcription.

*PGK1* encodes phosphoglycerate kinase, an enzyme that catalyzes an ATP-generating step of glycolysis, and its transcription is driven by a very strong TATA box-containing promoter [21–23]. This promoter was assigned as the “SAGA-dominated” [9] and “CR” [10] types, and thus its transcription is Taf1/TFIID-independent, when measured by steady-state mRNA levels [24–27]. In a previous study, we reported that *SPT3*, which encodes a TBP-regulatory SAGA subunit, facilitates *PGK1* transcription in a TATA box-dependent manner [25]. Interestingly, introduction of plasmid-borne *SPT3* into *spt3*Δ or *spt3*Δ *taf1* mutant strains rescued TATA box-dependent *PGK1* transcription but not Taf1/TFIID independence, particularly in transcription from a *PGK1* promoter lacking a TATA box [25]. However, the regulatory mechanism for this phenomenon, in which plasmid-borne *SPT3* failed to restore Taf1/TFIID independence, has yet to be identified.

In the present study, we first determined whether the gene location of *SPT3*, for example chromosomal or plasmid-borne, affected Taf1/TFIID-independent *PGK1* transcription, revealing that Taf1/TFIID dependence could be acquired regardless of gene location when Spt3 and Taf1 functions were compromised simultaneously during strain construction. Thus, the transcriptional mode of the *PGK1* promoter could be switched epigenetically from Taf1/TFIID-independent to Taf1/TFIID-dependent without altering the final yeast strain genotype. This was an unexpected finding, as most prior studies suggest that TFIID dependence or independence is determined by the promoter structure, including the upstream activating sequence (UAS), core promoter element, or their combination [24, 28–33]. Further analyses demonstrated that potential prion-like factor(s) generated in an Hsp104-dependent manner during strain construction affected the transcription mode not only of the *PGK1* promoter, but also of many other class II gene promoters. Based on these new findings, we suggest the intriguing possibility that TFIID and SAGA could form transcriptional condensates that support efficient transcription of a range of class II genes, and thereby that simultaneous functional loss of these two factors could alter the original traits of these condensates, switching the transcriptional mode from Taf1/TFIID-independent to Taf1/TFIID-dependent, even for the same promoter, without changing its primary structure.

## Materials and methods

### Yeast strains

Standard techniques were used for yeast growth and transformation [34]. Yeast strains used in the study are listed in S1 Table. Oligonucleotide sequences used for strain or plasmid construction are listed in S2 Table. All strains used in the study were generated from BY4741 or BY4742, as described below.

YTK16989/16993/16995/16999/17025/17029/17031/17035 have been described previously [25]. Briefly, while these strains carry a deletion of the chromosomal *TAF1*-coding region and either *TAF1* [YTK16989/16995/17025/17031] or the *taf1-N568Δ* [YTK16993/16999/17029/17035] gene in a *LEU2*-based low-copy vector, only YTK17025/17029/17031/17035 carry an additional deletion of the chromosomal *SPT3*-coding region. Furthermore, the strains carry a reporter *VTC1* gene on the chromosome that is driven by a TATA box-containing [YTK16989/16993/17025/17029] or TATA-less [YTK16995/16999/17031/17035] *PGK1* promoter.

YTK17039/17047/17051/17059 and YTK17037/17045/17049/17057 were generated from YTK17025/17029/17031/17035, respectively, by transformation with pM7932 (V5-tagged *SPT3*/pRS426) and pRS426 [35].

To generate YTK18751/18766/18778/18789, the four sub-fragments containing V5-tagged *SPT3* (primers: TK13236-TK13148/template: pM7932; hereafter abbreviated as TK13236-TK13148/pM7932), *ADH1* terminator [+1036 to +1235 bp] (TK13846-TK13856/pFA6a-13Myc-His3MX6 [36]), *URA3* (TK13510-TK13511/pM7932), or *SPT3* terminator [+1015 to +1227 bp] (TK13848-TK13849/BY4741), were fist amplified by PCR, and were then fused with TK13236-TK13849 to generate a 2.2-kb fragment that was used for transformation of YTK16989/16995/16993/16999, respectively.

To generate YTK20400/20413/20415/20425, the three sub-fragments containing the *SPT3* promoter [-489 to -1 bp] (TK13988-TK13989/BY4741), *TDH3* promoter [-673 to -24 bp] (TK13990-TK13991/BY4741), or V5-tagged *SPT3* + *URA3* (TK13987-TK13849/YTK18751), were first amplified by PCR, and then fused with TK13988-TK13849 to generate a 4.0-kb fragment that was used for transformation of YTK16989/16995/16993/16999, respectively.

YTK19261/19265/19267/19272 were generated from YTK17025/17031/17029/17035, respectively, by transformation with *Bal*I-linearized pM8169 (V5-tagged *SPT3*/pAUR101).

To generate YTK19100/19102/19105/19108, the 3.3-kb fragment containing V5-tagged *SPT3* + *ADH1* terminator + *URA3* (TK13750-TK13751/YTK18751), was first amplified by PCR, and then used for transformation of YTK17025/17029/17031/17035, respectively.

To generate YTK19228/19230, the 2.9-kb fragment containing the *VTC1* promoter [-320 to -1 bp] + loxP-*SpHIS5*-loxP [37] + *PGK1* promoter [-598 to -1 bp]+ *VTC1* ORF [+1 to +390 bp] + *VTC1* terminator [+391 to +603 bp] (TK7872-TK11973/pM8176 or pM8177), was first amplified by PCR, and was then used to transform YTK11411 [38], pYN1 [39] of which had been replaced with pM4201 (HA-tagged *TAF1*/pRS315) [25], respectively. Similarly, to generate YTK19236 and 19238, the same 2.9-kb fragment (TK7872-TK11973/pM8174 or pM8175) was amplified by PCR, and then used to transform YTK11406 [38], pYN1 of which had been replaced with pM4201.

YTK19244/19246/19252/19254 were generated from YTK19228/19230/19236/19238, respectively, by transformation with pSH47 [40], and then grown on galactose-containing synthetic media to induce *cre* recombinase expression, which resulted in excision of the *SpHIS5* marker located between the loxP sites.

YTK19281/19283 were generated from YTK19244/19246, respectively, by transformation with *Stu*I-linearized pM8165 (*TDH3* promoter + V5-tagged *SPT3* + *ADH1* terminator/pAUR101).

To generate YTK19301/19303, the three sub-fragments containing the *SPT3* promoter [-300 to -1 bp] (TK14415-TK14416/BY4741), *His3MX6* (TK4003-TK4006/pFA6a-13Myc-His3MX6 [36] or *SPT3* terminator [+1015 to +1287 bp](TK14417-TK14418/BY4741) were first amplified by PCR, and then fused with TK14415-TK14418 to generate a 2.7-kb fragment that was used for transformation of YTK19244/19246, respectively.

YTK19317/19319 were generated from YTK19301/19303, respectively, by transformation with *Stu*I-linearized pM8165.

To generate YTK19325/19327, the 2.7-kb fragment containing the *SPT3* promoter [-300 to -1 bp] + *His3MX6* + *SPT3* terminator [+1015 to +1287 bp] was amplified by PCR (TK14415-TK14418/ YTK19301) and used for transformation of YTK19281/19283, respectively.

YTK19349/19351/19357/19359 were generated from YTK19317/19319/19325/19327, respectively, by transformation with pYN1, and then by segregating out pM4201 (HA-tagged *TAF1*/pRS315).

YTK19401/19402/19405/19406 were generated from YTK19349/19351/19357/19359, respectively, by transformation with pM8201 (HA-tagged *taf1-N568Δ*/pRS315) [25], and then by shuffling out pYN1 on 5FOA-containing media.

YTK19397/19399 were generated from YTK19301/19303, respectively, by transformation with pYN1, and then by segregating out pM4201.

YTK19467/19469 were generated from YTK19397/19399, respectively, by transformation with pM8201, and then by shuffling out pYN1 on 5FOA-containing media.

YTK19489/19492 were generated from YTK19467/19469, respectively, by transformation with *Stu*I-linearized pM8165.

YTK19551/YTK19553/19587/19590 were generated from YTK19244/19246/19252/19254, respectively, by transformation with pYN1, and then by segregating out pM4201.

To create YTK19613/19614, the 2.7-kb fragment containing the *SPT3* promoter [-300 to -1 bp] + *His3MX6* + *SPT3* terminator [+1015 to +1287 bp] was amplified by PCR (TK14415-TK14418/ YTK19301), and used for transformation of YTK19587/19590, respectively.

YTK19623/19624 were generated from YTK19613/19614, respectively, by transformation with *Stu*I-linearized pM8165.

YTK19663/19665 were generated from YTK19623/19624, respectively, by transformation with pM4201, and then by shuffling out pYN1 on 5FOA-containing media.

YTK19626/19628/19664/19666 were generated from YTK19613/19614/19623/19624, respectively, by transformation with pM8201, and then by shuffling out pYN1 on 5FOA-containing media.

YTK19716/19717 were generated from YTK19626/19628, respectively, by transformation with *Stu*I-linearized pM8165.

YTK19489/19492/19317/19319/19489/19492/19401/19402/19401/19402 were crossed with YTK19664/19666/19663/19665/19716/19717/19664/19666/19716/19717, respectively, to generate YTK19746/19747/19748/19749/ 19763/19764/19765/19766/ 19767/19768, respectively.

YTK19746 and YTK19747 were sporulated and dissected to generate YTK19803/19804/19805/19806/19807/19808/19809/19810/19811/19812/19813/19814 and YTK19815/19816/19817/19818/19819/19820/19821/19822/19823/19824/19825/19826.

To generate YTK19981/19982/19983/19984, the three sub-fragments containing the *HSP104* promoter [-358 to -1 bp] (TK14581-TK14582/BY4741), *hphMX4* (TK14574-TK14575/pAG32 [41]), or 44 bp of the *KanMX4* linker *+ HSP104* terminator [+2728 to +3326 bp] (TK4716-TK14584/Y01514), were first amplified by PCR, and then were fused with TK14581-TK14584 to generate a 2.7-kb fragment that was used for transformation of YTK19551/19553/19587/19590.

To generate YTK19989/19990/19991/19992, the 2.7-kb fragment containing the *SPT3* promoter [-300 to -1 bp] + *His3MX6* + *SPT3* terminator [+1015 to +1287 bp]was amplified by PCR (TK14415-TK14418/YTK19301), and used for transformation of YTK19981/19982/19983/19984, respectively.

YTK20017/20018/20019/20020 were generated from YTK19989/19990/19991/19992, respectively, by transformation with *Stu*I-linearized pM8165.

YTK20037/20039/20041/20043 were generated from YTK20017/20018/20019/20020, respectively, by transformation with pM4201, and then by shuffling out pYN1 on 5FOA-containing media.

YTK20038/20040/20042/20044 were generated from YTK20017/20018/20019/20020, respectively, by transformation with pM8201, and subsequently by shuffling out pYN1 on 5FOA-containing media.

YTK20045/20046/20047/20048 were generated from YTK19989/19990/19991/19992, respectively, by transformation with pM8201, and then by shuffling out pYN1 on 5FOA-containing media.

YTK20049/20050/20051/20052 were generated from YTK20045/20046/20047/20048, respectively, by transformation with *Stu*I-linearized pM8165.

### Construction of plasmids

To create pM7932-expressing V5-tagged Spt3 in yeast cells, the three sub-fragments containing the *SPT3* promoter + *SPT3* ORF [-667 to +1011 bp](TK13229-TK13212/BY4741), V5 tag (TK6382-TK13148/ pOY0350, which is a 7xV5 tag-containing derivative of pM7318 [42]), or *SPT3* terminator [+1012 to +1227 bp](TK13213-TK13214/BY4741), were fist amplified by PCR, and then fused with TK13229-TK13214 to generate a 2.2-kb DNA fragment. This 2.2-kb fragment was digested with *Eco*RI and *Spe*I, and then ligated into the *Eco*RI/*Spe*I site of pRS426, generating pM7932.

The 2.2-kb fragment containing the *TDH3* promoter [-673 to -24] + V5-tagged *SPT3* ORF + *ADH1* terminator [+1036 to +1235 bp] (TK14327-TK14328/YTK20400) was amplified by PCR, digested with *Sph*I and *Xba*I, and then ligated into the *Sph*I/*Xba*I site of pAUR101 (TaKaRa), generating pM8165.

The 2.2-kb fragment containing the *SPT3* promoter [-667 to -1 bp] + V5-tagged *SPT3* ORF + *SPT3* terminator [+1015 to +1227 bp] (TK13985-TK13986/pM7932) was amplified by PCR, digested with *Sph*I and *Xba*I, and then ligated into the *Sph*I/*Xba*I site of pAUR101, generating pM8169.

To create pM8170/8171, the two sub-fragments containing the *PGK1* promoter [-598 to -1 bp]+ *VTC1* ORF [+1 to +190 bp](TK14391-TK14386/YTK16989 [TATA] or YTK16995 [GAGA]) or *VTC1* ORF [+168 to +390 bp]+ *VTC1* terminator [+391 to +690 bp](TK14385-TK2454/YTK16989 or YTK16995) were first amplified by PCR, and then fused with TK14391-TK2454 to generate a 1.3-kb DNA fragment, respectively. These 1.3-kb fragments ([TATA] or [GAGA]) were digested with *Bam*HI and *Xba*I, and then ligated into the *Bam*HI/*Xba*I site of pRS316 [43], generating pM8170/8171, respectively.

The 1.3-kb fragments containing the *PGK1* promoter [-598 to -1 bp] + *VTC1* [+1 to +690 bp](TK14391-TK2454/YTK16989 [TATA] or YTK16995 [GAGA]) were amplified by PCR, digested with *Bam*HI and *Xba*I, and then ligated into the *Bam*HI/*Xba*I site of pRS316, generating pM8172/8173, respectively.

To create pM8174/8175/8176/8177, the two sub-fragments containing *VTC1* [-764 to -1 bp] (TK14411-TK14389/BY4741) or loxP-*SpHIS5*-loxP (TK14387-TK14392/pLOXHIS5MS2L [44]) were first amplified by PCR, and were then fused with TK14411-TK14392 to generate 2.2-kb fragments. These 2.2-kb fragments were digested with *Bam*HI and *Hind*III, and then ligated into the *Bam*HI/*Hind*III site of pM8170/8171/8172/8173, generating pM8174/8175/8176/8177, respectively.

### Northern blot analysis

Northern analysis was performed as described previously [25]. For detection of *PGK1*, *VTC1*, and *SCR1*, DNA fragments were amplified by PCR from genomic DNA of BY4741, purified, and ^32^P-labeled by random priming with the Klenow fragment. The PCR primer pairs used were as described previously [25].

### Reverse transcription-quantitative PCR (RT-qPCR) analysis

Total RNA was isolated from the indicated strains grown to log-phase at 25ºC in synthetic complete media or further incubated at 37ºC for 2 h, as described previously [25]. cDNA synthesis was performed on 2 μg total RNA using random hexamers (Invitrogen) and Rever Tra Ace (Toyobo) according to the manufacturer’s protocol. RT-qPCR was performed on 1:5 diluted cDNA using a Light Cycler 96 system (Roche) with 1x THUNDERBIRD qPCR Mix (Toyobo) in a total reaction volume of 10 μL containing 0.3 μM of the appropriate primer pair. The results were analyzed using Light Cycler 96 Application Software Version 1.1 (Roche). After the PCR reaction, amplification specificity was confirmed by melt-curve analysis. The relative amounts of RNA in each sample were calculated for three independent biological replicates using the ΔCq method. The primer pairs used were as follows: *PGK1*, TK13695-TK13696; *VTC1*, TK13936-TK13937; *VTC1*^*#*^, TK14458-TK13937; *SCR1*, TK13934-TK13935.

### RNA-seq analysis

RNA levels of all genes in *TAF1* (YTK19317), *taf1-N568Δ* (YTK19401 [protocol #2], and YTK19489 [protocol #3]), *TAF1 hsp104*Δ (YTK20037), *taf1-N568Δ hsp104*Δ (YTK20038 [protocol #2] and YTK20049 [protocol #3]) strains cultured in the presence or absence of 3 mM Gdn-HCl were examined using RNA-seq analysis as described previously, with minor modifications [45]. Briefly, total RNA from each strain was prepared using ISOGEN with a Spin Column (Nippon Gene, Tokyo, Japan). cDNA sequencing libraries were generated from 1000 ng total RNA using a NEBNext Ultra II RNA Library Prep Kit for Illumina according to the manufacturer’s protocol. The libraries were amplified with seven PCR cycles. Pooled libraries were sequenced on an Illumina NextSeq 500 instrument, generating 75-bp single-end reads with 6-bp index tags. Analyses of sequenced data were conducted using CLC Genomics Workbench (QIAGEN) ver. 20.0.4. The single-end reads were trimmed to a fixed length of 35-bp from the 3’-end using the Trim Reads tool. Trimmed reads were mapped to the *S*. *cerevisiae* s288c reference genome (NC_001133–NC_001148) using the RNA-seq tool, with default parameters. Read data for RNA-seq experiments are accessible on DDBJ Sequence Read Archive under accession no. DRR403518-DRR403553. Clustering, volcano plot, and Venn diagram analyses were performed using in-house Python script.

## Results

### Taf1/TFIID dependence of *PGK1* transcription depends on the construction protocol of the *SPT3* *taf1-N568Δ* strain

A prior study demonstrated that Spt3 has two distinct functions on the *PGK1* promoter, TATA box-dependent 2–3-fold stimulation and conferring Taf1/TFIID independence of *PGK1* transcription [25]. Intriguingly, plasmid-borne non-tagged *SPT3* restored TATA box-dependent *PGK1* transcription, but not Taf1/TFIID-independent *PGK1* transcription, especially when the promoter TATA box was mutated to the GAGA sequence in the *spt3*Δ *taf1-N568Δ* strain [25]. Because *SPT3* expression driven by both low-copy and high-copy plasmids yielded similar results, specific loss of Taf1/TFIID-independent *PGK1* transcription was likely due to differences between chromosomal and plasmid-borne *SPT3* rather than simply to differences in Spt3 expression levels. Of note, Taf1/TFIID dependence of *PGK1* transcription was examined by the experimental procedure using a temperature shift from 25°C to 37°C, as the *taf1* strains used in our studies carried a temperature-sensitive *taf1-N568Δ* allele.

**Fig 1:**
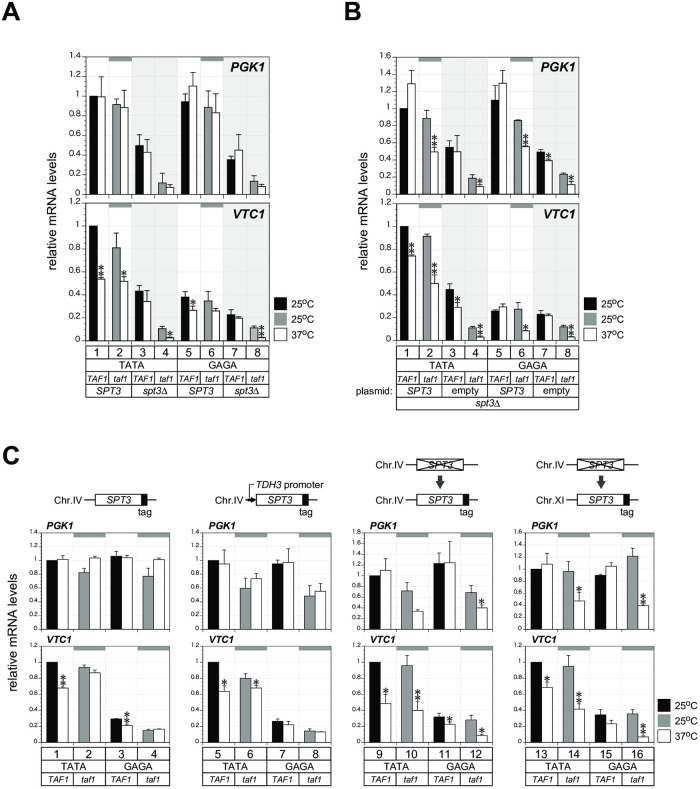
Taf1/TFIID dependence of *PGK1* transcription is dependent on the method used for *SPT3 taf1-N568Δ* strain construction. **(A)** RT-qPCR analyses to measure mRNA levels of *PGK1* (top panel) or *VTC1* (bottom panel) in the eight strains expressing the *VTC1* reporter driven by the TATA box-intact *PGK1* promoter (lanes 1–4) or the GAGA mutant (lanes 5–8), as indicated below the bottom panel. In these strains, chromosomal *TAF1* was deleted and then substituted with plasmid-encoded *TAF1* (odd-numbered lanes) or *taf1-N568Δ* (even-numbered lanes), while chromosomal *SPT3* was either intact (lanes 1, 2, 5, and 6) or deleted (lanes 3, 4, 7, and 8), as indicated below the bottom panel. The strains used are YTK16989 (lane 1), YTK16993 (lane 2), YTK17025 (lane 3), YTK17029 (lane 4), YTK16995 (lane 5), YTK16999 (lane 6), YTK17031 (lane 7), and YTK17035 (lane 8). Strains were cultivated at 25ºC and further incubated at 37ºC for 2 h in synthetic medium containing 2% glucose, as indicated at the right side of the *VTC1* panel. mRNA levels (mean ± SD of three biological replicates) were quantified and normalized to *SCR1* signal. All data are presented relative to that of lane 1 (25°C). The significance of differences between cultivation at 25°C and 37°C was assessed for each strain by t-test: * p < 0.05; ** p < 0.01. Reporter *VTC1* mRNA was more heat-sensitive than endogenous *PGK1* mRNA, even in the wild type strain (lane 1), as already shown by Northern blot analyses in a previous study [25]. The results from *spt3*Δ cells are shaded (lanes 3–4 and 7–8), while those obtained from *SPT3 taf1* cells are labeled with gray bars at the top of the graph (lanes 2 and 6). **(B)** RT-qPCR analyses to measure mRNA levels of *PGK1* (top panel) or *VTC1* (bottom panel) in the eight strains carrying the *VTC1* reporter driven by the *PGK1* promoter, in which the TATA box was intact (lanes 1–4) or substituted with the GAGA sequence (lanes 5–8), as indicated below the bottom panel. In these strains, chromosomal *TAF1* was deleted and substituted with plasmid-encoded *TAF1* (odd-numbered lanes) or *taf1-N568Δ* (even-numbered lanes), while chromosomal *SPT3* was deleted and then substituted with plasmid-encoded V5 epitope-tagged *SPT3* (lanes 1, 2, 5, and 6) or empty vector (lanes 3, 4, 7, and 8), as indicated below the bottom panel. Notably, *SPT3* expression plasmid or empty vector was introduced into *taf1*Δ *spt3*Δ strains carrying *TAF1* or *taf1-N568Δ* expression plasmids. The strains used were YTK17039 (lane 1), YTK17047 (lane 2), YTK17037 (lane 3), YTK17045 (lane 4), YTK17051 (lane 5), YTK17059 (lane 6), YTK17049 (lane 7), and YTK17057 (lane 8). Cultivation and data presentation were conducted as described in **A. (C)** RT-qPCR analyses to measure mRNA levels of *PGK1* (top panel) or *VTC1* (bottom panel) in the sixteen strains carrying the *VTC1* reporter driven by the *PGK1* promoter in which the TATA box was intact (lanes 1, 2, 5, 6, 9, 10, 13, and 14) or substituted with the GAGA sequence (lanes 3, 4, 7, 8, 11, 12, 15, and 16), as indicated below the bottom panel. The strains used in the first (lanes 1–4) or second (lanes 5–8) panel were generated by replacing chromosomal *SPT3* in the strains used in **A** (lanes 1, 2, 5, and 6) with V5 epitope-tagged *SPT3* driven by the *SPT3* promoter (lanes 1–4) or by the ectopic *TDH3* promoter (lanes 5–8). Similarly, the strains used in the third panel (lanes 9–12) or fourth panel (lanes 13–16) were generated by integrating V5 epitope-tagged *SPT3* driven by the *SPT3* promoter into the original *SPT3* locus (lanes 9–12) or the *AUR1* locus (lanes 13–16) of the strains used in **A** (lanes 3, 4, 7, and 8), respectively. The strains used were YTK18751 (lane 1), YTK18778 (lane 2), YTK18766 (lane 3), YTK18789 (lane 4), YTK20400 (lane 5), YTK20415 (lane 6), YTK20413 (lane 7), YTK20425 (lane 8), YTK19100 (lane 9), YTK19102 (lane 10), YTK19105 (lane 11), YTK19108 (lane 12), YTK19261 (lane 13), YTK19267 (lane 14), YTK19265 (lane 15), and YTK19272 (lane 16). Cultivation and data presentation were conducted as described in **A**.

To further explore the regulatory mechanism of this phenomenon, we employed the same *VTC1* reporter system used in a previous study [25], but quantified mRNA levels by RT-qPCR instead of Northern blot analysis. Using this reporter system, in which the endogenous *VTC1* promoter was replaced with test promoters, the transcriptional activities not only of modified or unmodified *PGK1* promoters, but also the transcriptional activity of the endogenous *PGK1* promoter, could be measured simultaneously in the same strain. Consistent with prior findings [25], plasmid-borne V5 epitope-tagged *SPT3* expressed from a high-copy plasmid failed to confer Taf1/TFIID independence to the *PGK1* promoter when the TATA box was mutated to GAGA (Fig 1A and 1B, lanes 6 [*VTC1*]. Note that the gene name in square parenthesis following lane number hereafter indicates the mRNA that is referred, for example [*PGK1*, *VTC1*] in the following sentence. Notably, *PGK1* transcription was also Taf1/TFIID-dependent with plasmid-borne *SPT3*, albeit weakly, even when the TATA box was intact (Fig 1A and 1B, lanes 2 [*PGK1*, *VTC1*] or lanes 6 [*PGK1*]).

Next, we determined if chromosomal V5 epitope-tagged *SPT3* could confer Taf1/TFIID dependence to the *PGK1* promoter, similar to plasmid-borne *SPT3*, when expressed at several-fold higher levels by the ectopic *TDH3* promoter. Chromosomal *SPT3* did not confer Taf1/TFIID dependence to the *PGK1* promoter, regardless of whether it was driven by the endogenous *SPT3* promoter (Fig 1C, lanes 2 and 4 [*PGK1*, *VTC1*]) or ectopic *TDH3* promoter (Fig 1C, lanes 6 and 8 [*PGK1*, *VTC1*]). Contrastingly, when V5 epitope-tagged *SPT3* was integrated into the original locus (Fig 1C, third panel) or the *AUR1* locus (Fig 1C, fourth panel) of the *TAF1 spt3*Δ or *taf1-N568Δ spt3*Δ strains used in lanes 3, 4, 7, and 8 of Fig 1A, *PGK1* transcription was Taf1/TFIID-dependent (Fig 1C, lanes 10, 12, 14, and 16 [*PGK1*, *VTC1*]) by comparison with the results described above (Fig 1C, lanes 2, 4, 6, and 8 [*PGK1*, *VTC1*]). Given that the *SPT3* strains tested in Fig 1B were also constructed from the same set of *spt3*Δ strains used in Fig 1A by transformation with an *SPT3* expression plasmid, Taf1/TFIID dependence of *PGK1* transcription was likely due to the construction protocol of the *SPT3 taf1-N568Δ* strain, rather than to the chromosomal or plasmid gene location of *SPT3*.

### Simultaneous functional loss of Spt3 and Taf1 during strain construction renders *PGK1* transcription Taf1/TFIID-dependent in the *SPT3* *taf1-N568Δ* strain

As described above, the *SPT3 taf1-N568Δ* strain, generated from the *spt3*Δ *taf1-N568Δ* strain by reintroduction of *SPT3* via either plasmid transformation [25] (Fig 1B) or chromosomal integration (Fig 1C, third and fourth panels), demonstrated that *PGK1* transcription was Taf1/TFIID-dependent, while *PGK1* transcription remained Taf1/TFIID-independent in the *SPT3 taf1-N568Δ* strain generated from a distinct *SPT3 taf1-N568Δ* strain by directly replacing *SPT3* via genomic recombination (Fig 1C, first and second panels). These observations suggest that Taf1/TFIID dependence could be conferred to the *PGK1* promoter in the *SPT3 taf1-N568Δ* strain only when Spt3 and Taf1 functions were compromised simultaneously during strain construction.

To confirm this finding, we examined the Taf1/TFIID dependence of *PGK1* transcription in three distinct *SPT3 taf1-N568Δ* strains carrying the same genetic backgrounds but generated by different genetic approaches (S1A Fig). Each strain construction protocol (#1, #2, or #3) was comprised of three common steps in different orders: (i) Chromosomal integration of *SPT3* into the *AUR1* locus (+*SPT3*), (ii) Deletion of *SPT3* by transformation with a *His3MX6* cassette (+*spt3*Δ), and (iii) replacement of *TAF1* with the *taf1-N568Δ* by plasmid shuffling (+shuffling). The orders of these three steps were [(i) +*SPT3*, (ii) +*spt3*Δ, (iii) +shuffling] for #1, [(i) +*spt3*Δ, (ii) +*SPT3*, (iii) +shuffling] for #2, and [(i) +*spt3*Δ, (ii) +shuffling, (iii) +*SPT3*] for #3 (S1A Fig). Note that the functions of Spt3 and Taf1 were compromised simultaneously only in protocol #3.

Consistent with above finding, RT-qPCR revealed that *PGK1* transcription was Taf1/TFIID-dependent only in the *SPT3 taf1-N568Δ* strain generated by protocol #3 (S1B Fig, lanes 9 and 10 [*PGK1*, *VTC1*]). This supports the conclusion that simultaneous functional loss of Spt3 and Taf1 during strain construction is a prerequisite for rendering *PGK1* transcription Taf1/TFIID-dependent in the *SPT3 taf1-N568Δ* strain.

### *Trans*-acting promoter reprogramming factor(s) are produced in the *SPT3* *taf1-N568Δ* strain experienced with simultaneous functional loss of Spt3 and Taf1 during strain construction

The observation that *PGK1* transcription was Taf1/TFIID-dependent in the *SPT3 taf1-N568Δ* strain specifically when it was generated by protocol #3 (S1 Fig), in which both Spt3 and Taf1 function were lost simultaneously during strain construction, raised the intriguing possibility that as-yet unidentified factors produced only under simultaneous loss of Taf1 and Spt3 could reprogram the transcription mode of the *PGK1* promoter from a Taf1/TFIID-independent state to a Taf1/TFIID-dependent state. To begin to evaluate this possibility, we sought to identify whether such promoter reprogramming factor(s) functioned *in cis* in a manner tightly associated with the originally reprogrammed *PGK1* promoter, or rather *in trans* in a more diffusible manner, for example even if the *PGK1* promoter was later introduced into cells.

**Fig 2:**
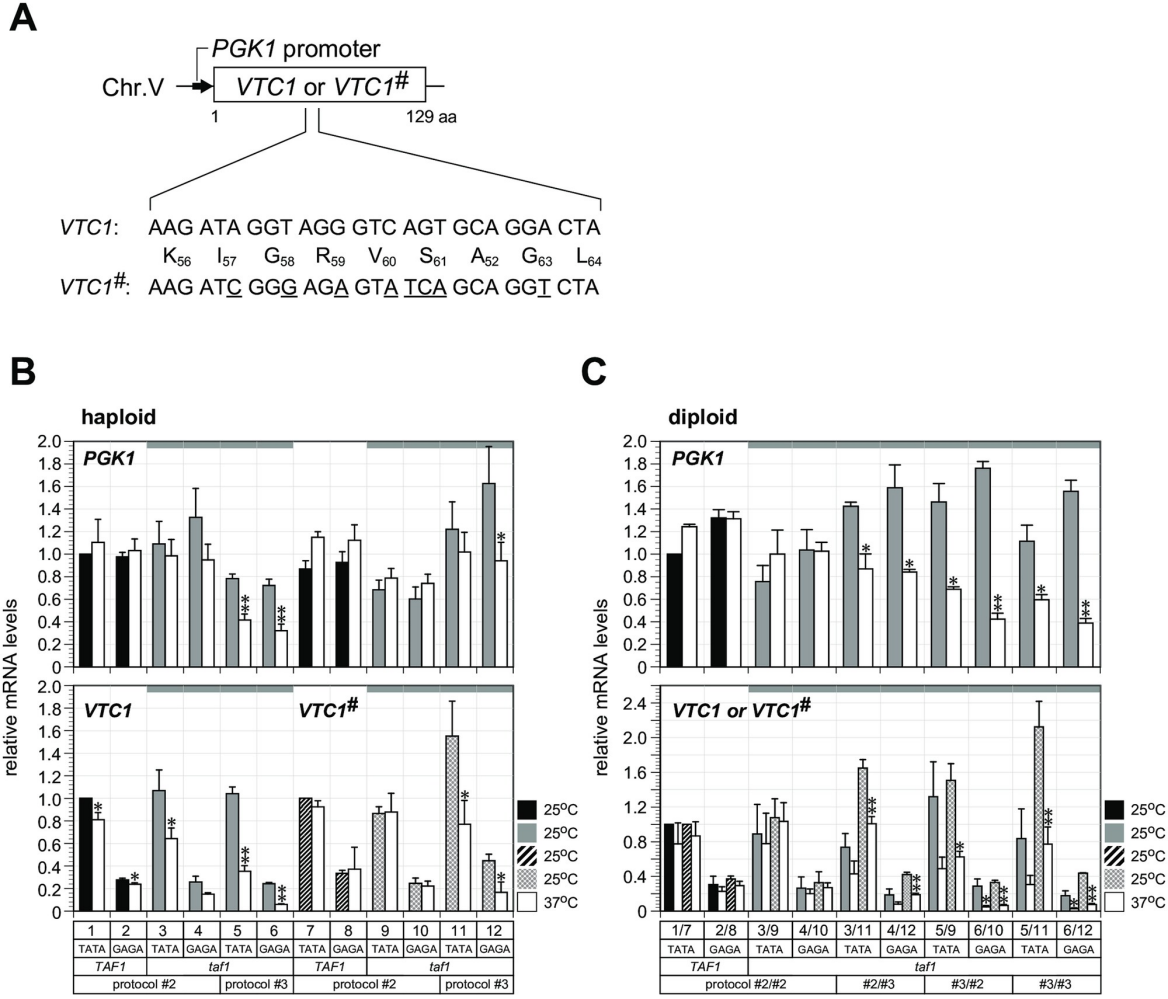
Examination of Taf1/TFIID dependence of *PGK1* transcription using the allele-specific *VTC1* reporter system. **(A)** Strains used in experiments to examine allele-specific expression of the *PGK1* promoter were constructed by replacing the *VTC1* reporter with the *VTC1*^*#*^ reporter carrying eight silent mutations (underlined). Promoter activities were analyzed by RT-qPCR using primers specific for each *VTC1* reporter allele. **(B)** RT-qPCR analysis to measure mRNA levels of *PGK1* (top panel) or *VTC1/VTC1*^*#*^ (bottom panel) in the twelve haploid strains carrying *VTC1* (lanes 1–6) or the *VTC1*^*#*^ reporter (lanes 7–12) driven by the *PGK1* promoter in which the TATA box was intact (odd-numbered lanes) or substituted with the GAGA sequence (even-numbered lanes), as indicated below the bottom panel. These strains were generated by construction protocol #2 (lanes 1–4 and 7–10) or #3 (lanes 5–6 and 11–12), as indicated below the bottom panel and described in S1A Fig. The strains used are YTK19317 (lane 1), YTK19319 (lane 2), YTK19401 (lane 3), YTK19402 (lane 4), YTK19489 (lane 5), YTK19492 (lane 6), YTK19663 (lane 7), YTK19665 (lane 8), YTK19664 (lane 9), YTK19666 (lane 10), YTK19716 (lane 11), and YTK19717 (lane 12). Cultivation and data presentation were conducted as described in Fig 1A. **(C)** RT-qPCR analysis to measure *PGK1* mRNA levels (top panel) or *VTC1/VTC1*^*#*^ mRNA levels (bottom panel) in the ten diploid strains carrying both of the *VTC1* and *VTC1*^*#*^ reporters driven by the *PGK1* promoter in which the TATA box was intact (lanes 1/7, 3/9, 3/11, 5/9, and 5/11) or substituted with the GAGA sequence (lanes 2/8, 4/10, 4/12, 6/10, and 6/12), as indicated below the bottom panel. These diploid strains YTK19748 (lanes 1/7), YTK19749 (lanes 2/8), YTK19765 (lanes 3/9), YTK19766 (lanes 4/10), YTK19767 (lanes 3/11), YTK19768 (lanes 4/12), YTK19746 (lanes 5/9), YTK19747 (lanes 6/10), YTK19763 (lanes 5/11), and YTK19764 (lanes 6/12), were obtained by crossing the two specific haploid strains listed in each lane of **B**. For instance, YTK19748 (lanes 1/7) was obtained by crossing YTK19317 (lane 1 in **B**) and YTK19663 (lane 7 in **B**). The other lanes are also numbered accordingly. Cultivation and data presentation were conducted as described in Fig 1A, except that the two sets of data measured individually for each reporter allele were presented together in one lane.

We thus generated a *VTC1*^#^ allele carrying eight silent point mutations in the middle of the open reading frame (ORF) (Fig 2A), such that the mRNA transcript of the *VTC1*^#^ allele could be distinguished from that of the original *VTC1* allele by RT-qPCR, as evidenced by the observation that specific amplification signals were not detected by quantitation software for cDNA samples derived from the *VTC1* strain. Specificity of the primer pairs used for RT-qPCR was also confirmed by conventional RT-PCR (S2 Fig). The *SPT3 taf1-N568Δ* strains harboring either *VTC1* or *VTC1#* were generated by protocol #2 or #3 and *PGK1* transcription was measured (Fig 2B). *PGK1* transcription was Taf1/TFIID-dependent in *SPT3 taf1-N568Δ* strains generated by protocol #3 (Fig 2B, lanes 5, 6, 11, and 12 [*PGK1*, *VTC1*, *VTC1*^*#*^]), but not in strains generated by protocol #2 (Fig 2B, lanes 3, 4, 9, and 10 [*PGK1*, *VTC1*, *VTC1*^*#*^]), regardless of which reporter alleles they harbored.

We subsequently measured *PGK1* transcription in diploid strains harboring both *VTC1* and *VTC1*^*#*^ alleles generated by crossing two of several haploid *SPT3 taf1-N568Δ* strains tested in Fig 2B in various combinations (Fig 2C). Remarkably, we found that *PGK1* transcription of both reporter alleles and presumably both endogenous *PGK1* alleles, while the latter two could not be discriminated by RT-qPCR, was Taf1/TFIID-dependent in the diploid strains when at least one mother haploid strain was generated by protocol #3 (Fig 2C, lanes 3/11, 4/12, 5/9, 6/10, 5/11, and 6/12 [*PGK1*, *VTC1*, *VTC1*^*#*^]). Thus, *PGK1* transcription remained Taf1/TFIID-independent only in the diploid strain in which both mother haploid strains were generated by protocol #2 (Fig 2C, lanes 3/9 and 4/10 [*PGK1*, *VTC1*, *VTC1*^*#*^]). These observations indicated that promoter reprogramming factor(s) produced in mother haploid strains generated by protocol #3 (i.e., those experienced with simultaneous functional loss of Spt3 and Taf1) functioned *in trans* to alter the transcription mode of a *PGK1* promoter that had not yet been reprogrammed.

### Potential prion-like properties of promoter reprogramming factor(s)

The reprogramming phenomenon observed for the *PGK1* promoter was likely to be an epigenetic event, as all the tested *SPT3 taf1-N568Δ* strains carried essentially the same genetic backgrounds, irrespective of whether *PGK1* transcription was Taf1/TFIID-independent or Taf1/TFIID-dependent. Thus, we subsequently examined the inheritance of this unexpectedly acquired trait from mother diploid strains (Fig 2C, lanes 5/9 and 6/10) to haploid progenitors (S3 Fig). This trait was inherited by progenitors evenly but somewhat weakly, as exemplified more evidently by the results obtained for progenitors carrying the *VTC1*^*#*^ reporter gene driven by the GAGA-substituted *PGK1* promoter (compare lanes 21–32 with lanes 4 and 8 in S3 Fig). This non-mendelian inheritance strongly supports the notion that this alteration of transcription mode was an epigenetic event, involving *trans*-acting and meiotically unstable promoter reprogramming factor(s), to render the *PGK1* promoter Taf1/TFIID-dependent.

To determine if these epigenetic promoter reprogramming factor(s) had prion-like properties, *PGK1* transcription was examined in haploid or diploid strains used in Fig 2 after cultivation for a further few days in media containing 3 mM of guanidine hydrochloride (Gdn-HCl) (S4 Fig). Gdn-HCl eliminates prions by inhibiting Hsp104 chaperone activity essential for prion heritability [46, 47]. However, the obtained results were more complicated than expected, as the Gdn-HCl treatment decreased Taf1/TFIID dependence of *PGK1* transcription in the *VTC1*-carrying haploid strain constructed by protocol #3 (Fig 2B and S4A Fig, lanes 5 and 6 [*VTC1*, *PGK1*]). However, and surprisingly, Gdn-HCl treatment increased Taf1/TFIID dependence of *PGK1* transcription in the *VTC1*-carrying haploid strain constructed by protocol #2 (Fig 2B and S4A Fig, lanes 3 and 4 [*VTC1*, *PGK1*]). Furthermore, in the *VTC1*^*#*^-carrying haploid strains, Taf1/TFIID dependence or Taf1/TFIID independence of *PGK1* transcription was not significantly affected by Gdn-HCl treatment, regardless of whether the strains were constructed by protocol #2 or #3 (Fig 2B and S4A Fig, lanes 9, 10, 11 and 12 [*VTC1*^*#*^, *PGK1*]). Contrastingly, in the diploid strains obtained from one or more haploid progenitors generated by protocol #3, Gdn-HCl treatment decreased Taf1/TFIID dependence of *PGK1* transcription, which was more evident for endogenous *PGK1* transcription than reporter gene transcription (Fig 2C and S4B Fig, lanes 3/11, 4/12, 5/9, 6/10, 5/11 and 6/12 [*VTC1*, *VTC1*^*#*^, *PGK1]*). Importantly, *PGK1* transcription in the diploid strain obtained by crossing two haploid strains, both of which were constructed by protocol #2, was nearly affected by Gdn-HCl treatment (Fig 2C and S4B Fig, lanes 3/9 and 4/10 [*VTC1*, *VTC1*^*#*^, *PGK1*]). Collectively, these observations suggest that the promoter reprogramming factor(s) could have prion-like properties, but the effect of Gdn-HCl-mediated HSP104 inhibition on their function was complex.

### Hsp104 plays an essential role in producing promoter reprogramming factor(s)

**Fig 3:**
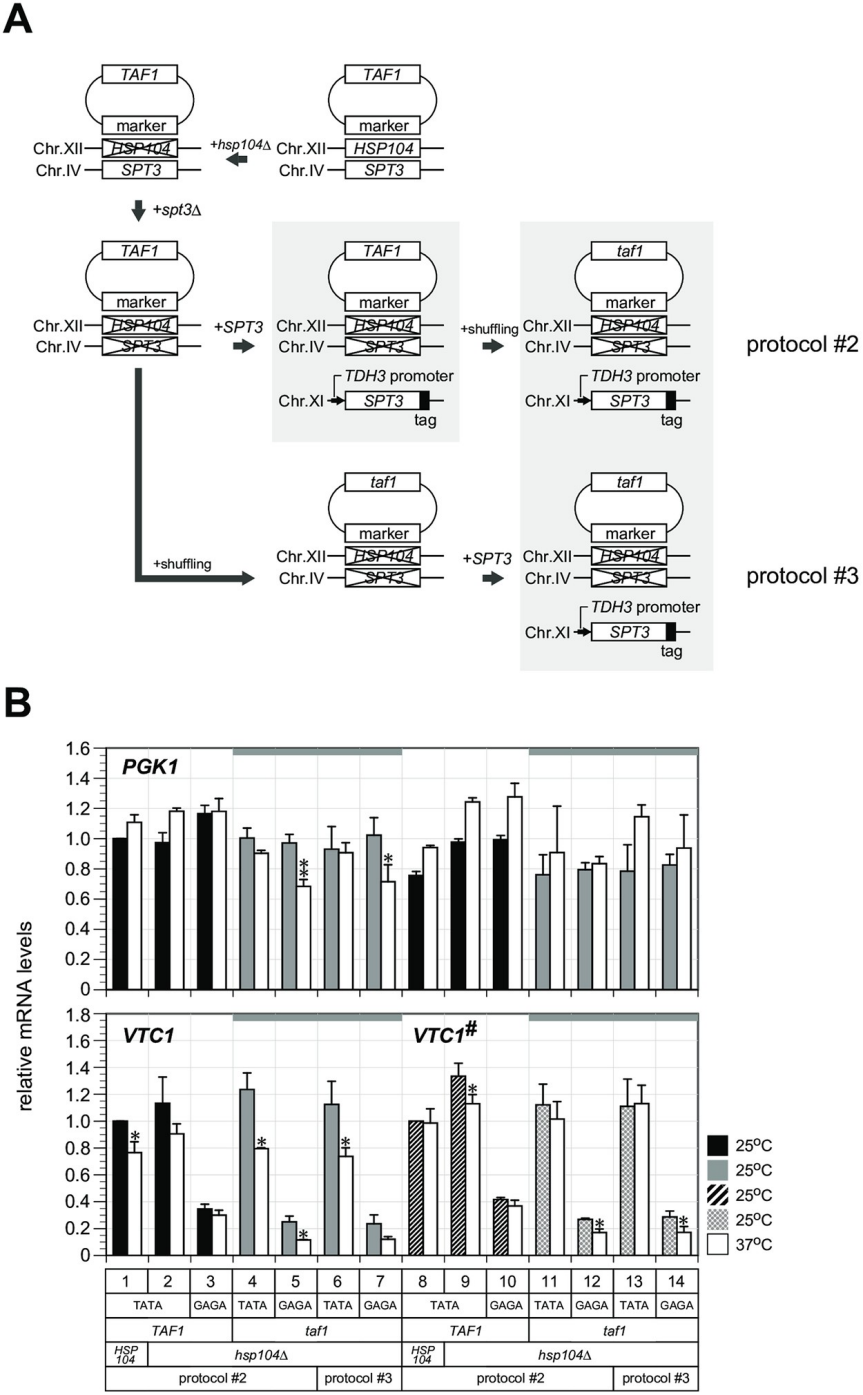
Effect of the *hsp104*Δ mutation on Taf1/TFIID dependence of the *PGK1* promoter via protocol #3. **(A)** Schematic outline of strain construction protocols #2 and #3 in strains harboring the *hsp104*Δ mutation. These protocols were the same as described in S1A Fig, except that the starting strain carried the *hsp104*Δ mutation. **(B)** RT-qPCR analysis to measure *PGK1* mRNA levels (top panel) or *VTC1/VTC1*^*#*^ mRNA levels (bottom panel) in the 14 haploid strains carrying *VTC1* (lanes 1–7) or the *VTC1*^*#*^ reporter (lanes 8–14) driven by the *PGK1* promoter in which the TATA box was intact (lanes 1, 2, 4, 6, 8, 9, 11, and 13) or substituted with the GAGA sequence (lanes 3, 5, 7, 10, 12, and 14), as indicated below the bottom panel. These strains were generated by protocol #2 (lanes 1–5 and 8–12) or #3 (lanes 6–7 and 13–14), respectively, as indicated below the bottom panel and as described in **A**. The strains used were YTK19317 (lane 1), YTK20037 (lane 2), YTK20039 (lane 3), YTK20038 (lane 4), YTK20040 (lane 5), YTK20049 (lane 6), YTK20050 (lane 7), YTK19663 (lane 8), YTK20041 (lane 9), YTK20043 (lane 10), YTK20042 (lane 11), YTK20044 (lane 12), YTK20051 (lane 13), and YTK20052 (lane 14).

Because we identified that Gdn-HCl inhibition of Hsp104 affected Taf1/TFIID dependence of the *PGK1* promoter, we next sought to examine more directly the effect of Hsp104 by genetic approaches utilizing the *hsp104*Δ mutation. For this purpose, test haploid strains were constructed by protocol #2 or #3 after disruption of the *HSP104* gene (Fig 3A). We did not detect significant differences in the *PGK1* transcription profiles between these two types of strains, regardless of whether they were constructed by protocol #2 or #3 (Fig 3B, lanes 4–7 [*VTC1*, *PGK1*] and 11–14 [*VTC1*^*#*^, *PGK1*]), indicating that Taf1/TFIID dependence could be conferred to the *PGK1* promoter specifically by protocol #3 only when Hsp104 function was intact. Because the *hsp104*Δ mutation did not affect *PGK1* transcription (compare the results of Figs 2B and 3B, based on those of lanes 1 obtained for the same strain), Hsp104 likely had an essential role in production of promoter reprogramming factor(s) but not for transcriptional regulation itself. Northern blot analyses using probes targeted for different regions of *PGK1* and *VTC1* ORFs from those amplified by the RT-qPCR primers (S5 Fig) were consistent with RT-qPCR findings, minimizing the possibility that the findings were due to unexpected artifacts such as transcript sequence variance.

### Genome-wide RNA-seq analyses of strains constructed by protocol #2 or #3 in the presence or absence of Hsp104 inhibition or mutation

The above analyses indicated that promoter reprogramming factor(s) that switch the transcription mode of the *PGK1* promoter could be produced in *SPT3 taf1-N568Δ* strains constructed by protocol #3 in an Hsp104-dependent manner. To examine how broadly these factor(s) could reprogram the transcription mode of other class II genes as well as how extensively this reprogramming could be affected by Gdn-HCl treatment or the *hsp104*Δ mutation, we conducted genome-wide RNA-seq analyses for the *SPT3 TAF1* (wild type [WT] as a control) and *SPT3 taf1-N568Δ* strains constructed by protocol #2 or #3 (hereafter designated as #2 strains or #3 strains) in the presence or absence of Gdn-HCl treatment or the *hsp104*Δ mutation.

**Fig 4:**
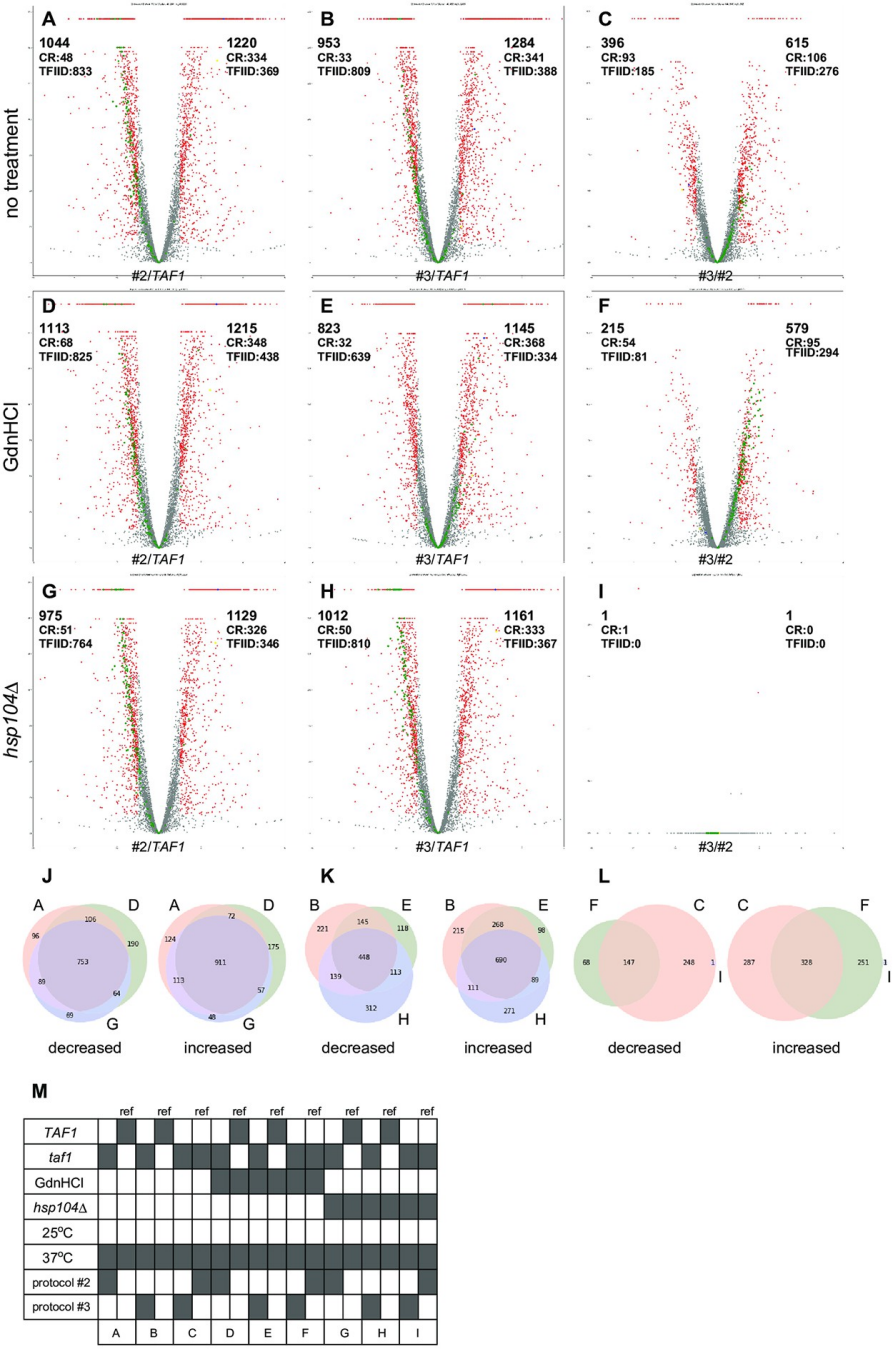
Volcano plot analyses of the gene expression profiles of #2 and #3 strains reveal *HSP104* dependency of differential gene expression. TPM data of the RNA-seq analyses obtained from two biological replicates of six strains with and without *taf1* or *hsp104*Δ mutations after cultivation under various conditions, which are indicated in the bottom panel, were averaged and subjected to volcano plot analyses. Fold changes of gene expression in the #2 strain or #3 strain relative to that in the *TAF1* strain are shown in A and B, respectively. Similarly, fold changes of gene expression in the #3 strain relative to that in the #2 strain are shown in C. The analogous three sets of comparisons were conducted for the TPM data derived from strains cultivated in the presence of Gdn-HCl (D, E, F) or the *hsp104*Δ mutation (G, H, I). In A–I, the genes considered to be significantly differentially expressed (> 2-fold change, p < 0.05) are indicated with red dots, while ribosomal protein genes, ~95% of which are TFIID-dependent genes [10], are indicated with green dots. Furthermore, the numbers of class II genes or those of the subclasses CR or TFIID-dependent, that were significantly differentially expressed, are shown in the upper left or upper right corners of A–I. The overlaps among these differentially expressed class II genes across A, D, and G (#2 strain versus *TAF1*), B, E, and H (#3 strain versus *TAF1*) and C, F, and I (#3 strain versus #2 strain) are visualized by Venn diagrams in J, K, and L, respectively. The cultivation conditions and comparison details for A-I are summarized in M. The strains used were YTK19317 (A, B, D, E), YTK19401 (A, C, D, F), YTK19489 (B, C, E, F), YTK20037 (G, H), YTK20038 (G, I), and YTK20049 (H, I). Cultivation was performed as described in Fig 1A and S4 Fig.

As expected, volcano plot analyses of TPM (transcripts per million) data revealed that the gene expression profiles of the #2 and #3 strains were significantly different from that of the WT strain at 37°C (Fig 4A and 4B) and even at 25°C (S6A and S6B Fig). Importantly, the gene expression profiles of the #3 strains were significantly different from those of the #2 strains, regardless of whether the strains were incubated at 37°C (Fig 4C and 4F) or 25°C (S6C and S6F Fig) in the absence (Fig 4C, S6C Fig) or presence (Fig 4F, S6F Fig) of Gdn-HCl. In greater contrast, we found that the gene expression profiles of the entire set of genes were nearly the same for the #2 and #3 strains under the presence of the *hsp104*Δ mutation when they were incubated at 37°C (Fig 4I) or 25°C (S6I Fig). These observations suggest that promoter reprogramming factor(s), which could be produced specifically in the #3 strains in an Hsp104-dependent manner, affect the transcription mode not only of the *PGK1* promoter but also of many other class II gene promoters.

Two distinct types of genes in budding yeast were recently distinguished, which are designated as "coactivator-redundant (CR)" and "TFIID-dependent" genes [10]. Transcription of CR genes is modestly affected by rapid depletion of either TFIID or SAGA singly, but is severely affected by simultaneous depletion of TFIID and SAGA. On the other hand, transcription of TFIID-dependent genes is severely affected by rapid depletion of TFIID alone, and is only modestly affected by SAGA depletion [10]. Because *PGK1* is a CR gene [10], we examined the possibility that differences of gene expression levels between #2 strains and #3 strains could be more significant for CR genes than for TFIID-dependent genes. To test this hypothesis, the numbers of affected genes (criteria: > 2-fold change, p < 0.05) were counted not only for the entire set of genes, but also for the two types of genes separately (Fig 4A–4I, S6A–S6I Fig). The lists of these counted genes are summarized in S3 Table (37°C) and 4 (25°C). Among the total 655 CR genes, the percentages of differentially expressed CR genes between #2 strains and #3 strains were greater than that of similarly affected differentially expressed (> 2-fold change, p < 0.05) TFIID-dependent genes (4245 genes total), regardless of incubation temperature or Gdn-HCl treatment (Fig 4C and 4F and S6C and S6F Fig). Thus, CR genes were likely more sensitive to the effects of the promoter reprogramming factor(s) than TFIID-dependent genes.

Further, Venn diagrams revealed that the differentially expressed genes between WT and #2 strains (Fig 4A, 4D and 4G) were not as significantly affected by Gdn-HCl treatment or *hsp104*Δ mutation, as the differentially expressed genes between WT and #3 strains (Fig 4B, 4E and 4H), at least when the strains were incubated at 37°C (Fig 4J and 4K). This indicated that Gdn-HCl treatment could alter the properties of promoter reprogramming factor(s) in a manner different from that of the *hsp104*Δ mutation, which is consistent with its complex effects on the *PGK1* promoter (S4 Fig).

Finally, hierarchical clustering analysis for the same TPM data (S7 Fig) revealed that the *PGK1* promoter driving expression of *VTC1* reporter gene as well as endogenous *PGK1* was grouped into a subcluster comprising the 21 most highly expressed genes (S8A Fig), which was visualized at the top of the heatmap (S7 Fig). Intriguingly, 12 out of the 21 most highly expressed genes were related to the processes of either glycolysis or ethanol fermentation (S8B Fig), suggesting that promoters of glycolytic and fermentative genes could be regulated by a common mechanism that was similarly affected by several distinct genetic alterations (*taf1*, *spt3*Δ, *hsp104*Δ), by the order of mutation introduction during strain construction and/or physicochemical perturbations such as Gdn-HCl or increased temperature.

## Discussion

In the present study, we demonstrated that the transcription mode of the *PGK1* promoter could be switched from TFIID-independent to TFIID-dependent by as-yet unidentified promoter reprograming factor(s) produced specifically in yeast strains that underwent simultaneous functional loss of Taf1 and Spt3 during their construction. Intriguingly, production of these factor(s) was Hsp104-dependent, and affected transcription not only of the *PGK1* promoter but also those of many other class II genes, including coactivator-redundant (CR) and TFIID-dependent genes.

Hsp104 plays an essential role in propagation of the amyloid fiber-forming type of prions by several distinct mechanisms, including increased number of prion seeds due to its severing activity [48]. On the other hand, a recently identified non-amyloid type of prion, which is primarily comprised of nucleic acid binding proteins with large intrinsically disordered domains (IDDs), can be propagated via Hsp104, Hsp70, Hsp90, or other chaperones [49]. The present observation that the promoter reprograming trait was genetically dominant and inherited to progenitors in a non-mendelian fashion implies that the promoter reprogramming factor(s) could potentially be either a type of prion, albeit with some unconventional properties such as meiotic instability and a complex response to Gdn-HCl treatment.

Recent studies have demonstrated that many components of the transcription machinery, including the sequence-specific transcriptional regulators, Med1, p300, Brd4, P-TEFb, and RNA polymerase II, can form transcriptional condensates individually or collectively, which are membrane-less compartments built up with RNA and/or DNA in the nucleus to support efficient transcription via a liquid-liquid phase separation mechanism [17, 50–52]. Because cytoplasmic biomolecular condensates such as stress granules [53] or heat-inducible condensates [54] require Hsp104 for their dynamic behaviors, the phase transition of some nuclear transcriptional condensates could also be mediated by these chaperone functions. If so, another possibility is that the promoter reprogramming factor(s) might not be a prion form of particular protein(s), but rather reflect a certain phase-transitioned state of the transcriptional machinery that is induced by the simultaneous functional loss of Taf1 and Spt3 during strain construction. To further explore these possibilities, identification of causal or involved protein(s) as well as examination of their physical properties would be required, especially considering that proteins such as Sup35 [55] and p53 [56] form not only amyloid fibrils but also biomolecular condensates.

One of the most remarkable observations in the study was that the genome-wide gene expression profile of the #3 strain was very similar to that of the #2 strain under the presence of the *hsp104*Δ mutation when cultured at either 37°C (Fig 4I) or 25°C (S6I Fig). On the other hand, the genome-wide gene expression profile of the #2 strain was significantly altered by the *hsp104*Δ mutation at both temperatures (Fig 4J and S6J Fig), indicating that Hsp104 plays a crucial role not only in generating promoter reprograming factor(s) but also in maintaining the original gene expression profile of the #2 strain. Furthermore, it should be noted that the difference of the gene expression profile between the WT and #2 strains was significantly diminished by the presence of the *hsp104*Δ mutation in culture at 25°C (S6G Fig). This implies that Hsp104 could allow #2 strain to acquire a unique gene expression profile under permissive growth conditions, presumably via the aid of either prion(s) or condensate(s) that would be generated ectopically in the #2 strain, even in which the functional losses of TFIID and SAGA were minimal and null, respectively. Importantly, the identity of the factors generated in the #2 strain and their relationship to the promoter reprograming factor(s) generated in the #3 strain are unclear. In any case, these observations raised an intriguing possibility that the altered gene expression profiles obtained at restrictive temperatures for the *taf1-N568Δ* mutant (#2 strain) as well as for many other previously reported *taf* mutants [24, 57–72] could be achieved by the combined effects not only of impaired TFIID function but also of other prion(s) or condensate(s) ectopically generated in *taf* mutant strains.

TFIID and SAGA both play important roles in transcription of nearly all class II genes [10, 73, 74]. Prior studies suggest that TBP loading for TFIID-dependent genes is mediated by TFIID, while TBP loading for CR genes is mediated redundantly by TFIID and SAGA [10]. Furthermore, chromatin modifications established by SAGA and eliminated slowly after SAGA depletion are important for transcription of both TFIID-dependent and CR genes [10]. These views were obtained by the studies using ChEC-seq and/or nascent transcript labeling techniques instead of more traditional ChIP-seq and/or steady-state transcript analyses. The amounts of steady-state mRNAs do not accurately reflect the activity of ongoing transcription, especially in yeast cells defective of pol II transcriptional machinery, including TFIID and SAGA, as these transcriptional defects are often masked by cytoplasmic stabilization of the corresponding mRNAs [73–77]. This “transcript buffering” effect [77] could explain the apparently limited effects of some TFIID and SAGA mutations on global transcription assessed by measurement of steady-state mRNAs. Therefore, the transcription mode of some genes could be revealed as being altered from TFIID-independent to TFIID-dependent, similar to that of the *PGK1* promoter, if the transcript buffering system becomes dysfunctional and thereby unable to compensate for impaired TFIID activity. Considering that the Ccr4-Not complex plays a crucial role in cytoplasmic transcript buffering [77] and interacts genetically with TFIID and SAGA [78, 79], this is a potential candidate for the aforementioned promoter reprograming factor(s). Consistent with this, recent studies demonstrated that Not1 [80] and Not5 [81] form novel biomolecular condensates to enable elaborate translational regulation, while other subunits of the Ccr4-Not complex such as Not2, Ccr4, and Pop2, are components of the processing bodies [82]. Furthermore, the liquid-like state of the processing bodies was maintained in an Hsp104-dependent manner [83]. To confirm whether the Ccr4-Not complex is a true promoter reprograming factor, the effect of gene disruption of this complex on Taf1/TFIID dependence of the *PGK1* promoter would need to be evaluated in a strain generated similarly to the #3 strain in the present study. In any case, identification of promoter reprograming factor(s) would provide important insights into understanding how prions or biomolecular condensates are involved in TFIID- and SAGA-mediated gene transcription.

## Supporting information

S1 Fig Simultaneous functional loss of Spt3 and Taf1 during the strain construction process conferred Taf1/TFIID dependence to the *PGK1* promoter. **(A)** Schematic outline of the three strain construction protocols (#1, #2, and #3) used to generate the *SPT3*/*TAF1* or *SPT3/ taf1-N568Δ* strains. These three protocols are all comprised of three common steps: (i) chromosomal integration of *SPT3* into the *AUR1* locus driven by the ectopic *TDH3* promoter (+*SPT3*), (ii) deletion of *SPT3* by transformation with a *His3MX6* cassette (+*spt3*Δ), and (iii) replacement of *TAF1* with *taf1-N568Δ* by plasmid shuffling (+shuffling). Note that these three steps are conducted in a different order in each protocol: #1 [(i) +*SPT3*, (ii) +*spt3*Δ, (iii) +shuffling], #2 [(i) +*spt3*Δ, (ii) +*SPT3*, (iii) +shuffling], and # 3 [(i) +*spt3*Δ, (ii) +shuffling, (iii) +*SPT3*]. The two *TAF1* and three *taf1* strains with the same genetic backgrounds are shaded for clarification. **(B)** RT-qPCR analyses to measure *PGK1* (top panel) or *VTC1* (bottom panel) mRNA levels in the ten strains carrying the *VTC1* reporter driven by the *PGK1* promoter in which the TATA box was intact (lanes 1, 2, 5, 6, and 9) or substituted with the GAGA sequence (lanes 3, 4, 7, 8, and 10), as indicated below the bottom panel. Lane 1–4 strains were generated by protocol #1, lane 5–8 strains were generated by protocol #2, and lane 9–10 strains were generated by protocol #3, as indicated in **A**. The strain used are YTK19325 (lane 1), YTK19405 (lane 2), YTK19327 (lane 3), YTK19406 (lane 4), YTK19317 (lane 5), YTK19401 (lane 6), YTK19319 (lane 7), YTK19402 (lane 8), YTK19489 (lane 9), and YTK19492 (lane 10). Cultivation and data presentation were conducted as described in Fig 1.
(TIF)

S2 Fig cDNA was amplified allele-specifically by conventional RT-PCR using the same primer pairs as those used for RT-qPCR studies. PCR was conducted using cDNA derived from YTK19317 (lanes 1, 2) or YTK19663 (lanes 3, 4) as a template, and primer pairs were *VTC1*-specific TK13936-TK13937 (lanes 1, 3) or *VTC1*^*#*^-specific TK14458-TK13937 (lanes 2, 4). These cDNA and primer pairs are the same as those used in lanes 1 and 7 of Fig 2B. PCR products were resolved by agarose gel electrophoresis and subsequently visualized by ethidium bromide staining, together with a size marker (MW, ExcelBand 1kb DNA ladder, SMOBIO).
(TIF)

S3 Fig Inheritance from mother diploid strains to haploid progenitors was tested for Taf1/TFIID dependence of *PGK1* transcription acquired epigenetically during the strain construction via protocol #3. RT-qPCR analysis to measure mRNA levels of *PGK1* (top panel) or *VTC1/VTC1*^*#*^ (bottom panel) in the 32 haploid strains carrying *VTC1* (lanes 1–4, 9–10, 13–14, 17–18, 21–22, 25–26, and 29–30) or the *VTC1*^*#*^ reporter (lanes 5–8, 11–12, 15–16, 19–20, 23–24, 27–28, and 31–32) driven by the *PGK1* promoter in which the TATA box was intact (lanes 1, 3, 5, 7, and 9–20) or substituted with the GAGA sequence (lanes 2, 4, 6, 8, and 21–32), as indicated below the bottom panel. The strains used in lanes 1–8 were the same as those used in lanes 1, 2, 3, 4, 7, 8, 9, and 10 of Fig 2B, respectively, as indicated. The other strains used in lanes 9–20 and 21–32 were haploid progenitors obtained by tetrad dissection of the mother #3/#2 diploid strains used in lanes 5/9 and 6/10 of Fig 2C, respectively, as indicated. The strains used are YTK19317 (lane 1), YTK19319 (lane 2), YTK19401 (lane 3), YTK19402 (lane 4), YTK19663 (lane 5), YTK19665 (lane 6), YTK19664 (lane 7), YTK19666 (lane 8), YTK19803 (lane 9), YTK19805 (lane 10), YTK19804 (lane 11), YTK19806 (lane 12), YTK19807 (lane 13), YTK19808 (lane 14), YTK19809 (lane 15), YTK19810 (lane 16), YTK19813 (lane 17), YTK19814 (lane 18), YTK19811 (lane 19), YTK19812 (lane 20), YTK19817 (lane 21), YTK19818 (lane 22), YTK19815 (lane 23), YTK19816 (lane 24), YTK19819 (lane 25), YTK19821 (lane 26), YTK19820 (lane 27), YTK19822 (lane 28), YTK19823 (lane 29), YTK19825 (lane 30), YTK19824 (lane 31), and YTK19826 (lane 32). Cultivation and data presentation were conducted as described in Fig 1A.
(TIF)

S4 Fig Effect of Gdn-HCl treatment on Taf1/TFIID dependence of *PGK1* transcription in haploid or diploid *SPT3 taf1-N568Δ* strains constructed by protocol #2 or #3. **(A)** RT-qPCR analysis to measure mRNA levels of *PGK1* (top panel) or *VTC1/VTC1#* (bottom panel) in the twelve haploid strains carrying *VTC1* (lanes 1–6) or the *VTC1*^*#*^ reporter (lanes 7–12) driven by the *PGK1* promoter in which the TATA was intact (odd-numbered lanes) or substituted with the GAGA sequence (even-numbered lanes), as indicated below the bottom panel. These strains are the same as those used in Fig 2B. Cultivation and data presentation were conducted as described in Fig 1A, except that the media used here contained 3 mM Gdn-HCl. **(B)** RT-qPCR analysis to measure mRNA levels of *PGK1* (top panel) or *VTC1/VTC1*^*#*^ (bottom panel) in the ten diploid strains carrying both of the *VTC1* and *VTC1*^*#*^ reporters driven by the *PGK1* promoter in which the TATA box was intact (lanes 1/7, 3/9, 3/11, 5/9, and 5/11) or substituted with the GAGA sequence (lanes 2/8, 4/10, 4/12, 6/10, and 6/12), as indicated below the bottom panel. The strains are the same as those used in Fig 2C. Cultivation and data presentation were conducted as described in Fig 1A, except that the media also contained 3 mM Gdn-HCl.
(TIF)

S5 Fig Effect of the *hsp104*Δ mutation on Taf1/TFIID dependence of *PGK1* transcription, as examined by Northern blot analysis. **(A)** Northern blot analysis of *VTC1* (middle panels), *PGK1* (top panels), or *SCR1* (control; bottom panels) RNA levels in the twelve strains used in Figs 2 (lanes 1–6) and 3 (lanes 7–12). The strains used are YTK19317 (lane 1), YTK19319 (lane 2), YTK19401 (lane 3), YTK19402 (lane 4), YTK19489 (lane 5), YTK19492 (lane 6), YTK20037 (lane 7), YTK20039 (lane 8), YTK20038 (lane 9), YTK20040 (lane 10), YTK20049 (lane 11), and YTK20050 (lane 12). Cultivation was performed as described in Fig 1A. **(B)** Raw expression data in **A** were quantified and normalized to *SCR1*. Values for each transcript derived from *PGK1* or *VTC1* are summarized in the upper or lower panel, respectively. In each panel, data are presented relative to the value obtained for the strain indicated on the left.
(TIF)

S6 Fig Volcano plot analyses for the TPM data obtained from strains cultured at 25°C. TPM data of RNA-seq analyses obtained from two biological replicates of six strains carrying or not carrying *taf1* or *hsp104*Δ mutations were averaged and subjected to volcano plot analyses as shown in Fig 4. Note that the data analyzed in this figure are derived from strains cultured at 25°C, while those analyzed in Fig 4 of the main text are derived from the strains cultured at 37°C. The analyzed data (A–L) and cultivation/comparison details (M) are represented as described in Fig 4.
(TIF)

S7 Fig Hierarchical clustering analyses of genome-wide expression of *TAF1* and *taf1* (#2, #3) strains in the presence or absence of Gdn-HCl treatment or the *hsp104*F044 mutation. Duplicate TPM data from six strains carrying or not carrying *taf1* or *hsp104*Δ mutations after cultivation under the conditions indicated in the bottom panel were subjected to hierarchical clustering analysis and visualized as a heat map. The same data were analyzed by volcano plot in Fig 4 and S6 Fig. Ribosomal protein genes (yellow), coactivator-redundant (CR) genes (red), and TFIID-dependent genes (blue) are indicated on the left side of the heatmap. Note that the gene list indicated on the right side of the heatmap is incomplete due to space limitations. The strains used are YTK19317 (columns #15, 18–20, 23–24, and 26–27), YTK19401 (columns #1–4 and 28–31), YTK19489 (columns #9–14 and 21–22), YTK20037 (columns #16–17, 25, and 32), YTK20038 (column #6–7, 33, and 35), and YTK20049 (columns #5, 8, 34, and 36). Cultivation was performed as described in Fig 1A and S4 Fig.
(TIF)

S8 Fig The majority of the 21 most highly expressed genes encode glycolytic or ethanol fermentation enzymes. The cluster comprising the 21 genes and located at the top of the heatmap in S7 Fig is enlarged for detail. Note that the *SPT3* and *VTC1* genes are driven by the *TDH3* and *PGK1* promoters, respectively, in the strains subjected to RNA-seq analyses. **(A)** The metabolic pathways from glucose to ethanol (glycolysis and ethanol fermentation) are summarized with the names of gene products (open rectangles) catalyzing the corresponding reactions, if they are present in the cluster shown in **A**.
(TIF)

S1 Table The list of *Saccharomyces cerevisiae* strains used in this study. (XLSX)

S2 Table The list of oligonucleotides used for strain or plasmid construction in this study. (XLSX)

S3 Table The list of the affected genes (criteria: > 2-fold change, p < 0.05) that are counted in Fig 4A–4I. (XLSX)

S4 Table The list of the affected genes (criteria: > 2-fold change, p < 0.05) that are counted in S6A–S6I Fig. (XLSX)
