# Supplementary figures and images for: TFIID dependency of steady-state mRNA transcription altered epigenetically by simultaneous functional loss of Taf1 and Spt3 is Hsp104-dependent
Source: PLoS One. 2023 Feb 9;18(2):e0281233. doi: 10.1371/journal.pone.0281233 (PMC9910645; doi:10.1371/journal.pone.0281233)

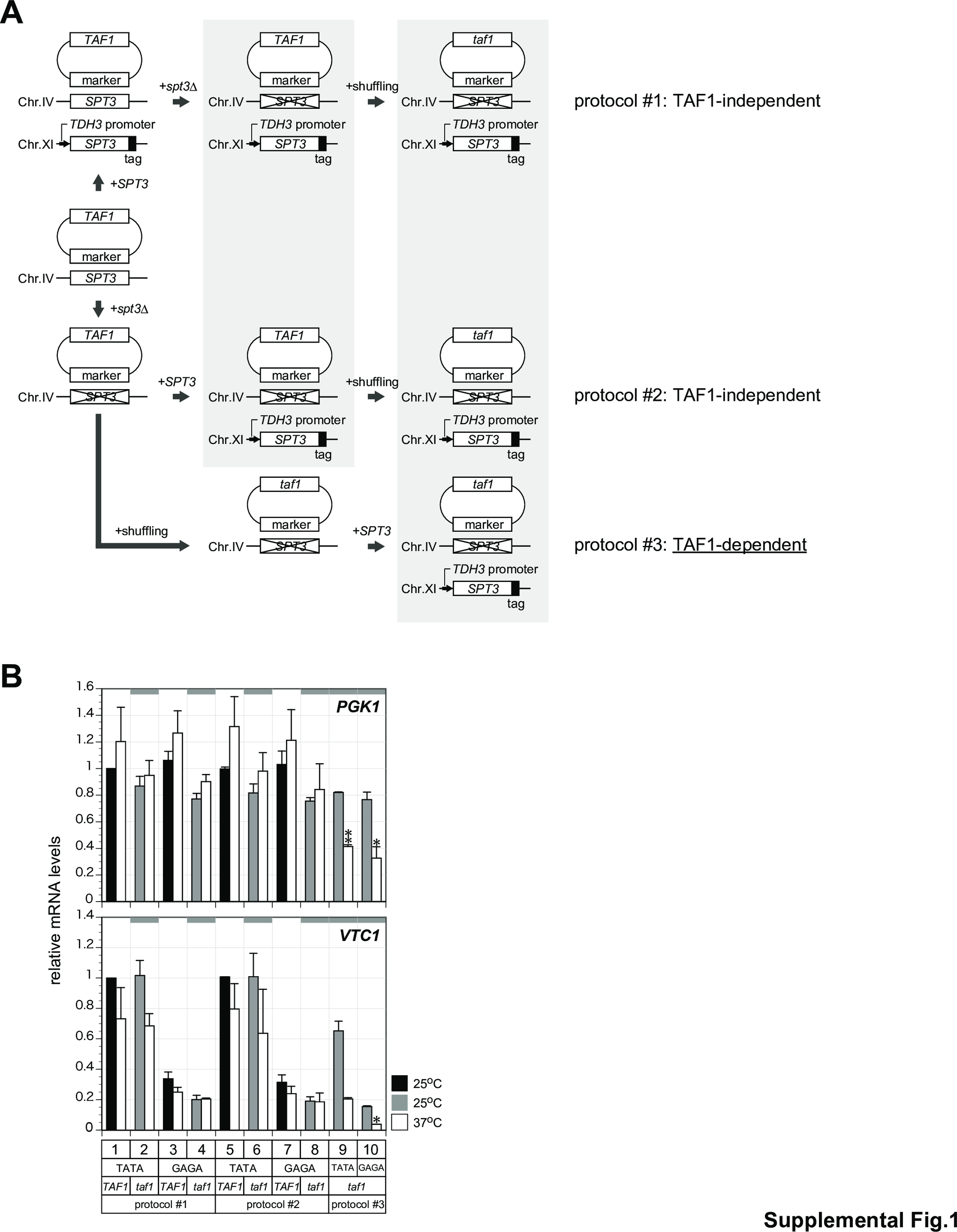

Supplement: S1 Fig — (A) Schematic outline of the three strain construction protocols (#1, #2, and #3) used to generate the SPT3/TAF1 or SPT3/ taf1-N568Δ strains. These three protocols are all comprised of three common steps: (i) chromosomal integration of SPT3 into the AUR1 locus driven by the ectopic TDH3 promoter (+SPT3), (ii) deletion of SPT3 by transformation with a His3MX6 cassette (+spt3Δ), and (iii) replacement of TAF1 with taf1-N568Δ by plasmid shuffling (+shuffling). Note that these three steps are conducted in a different order in each protocol: #1 [(i) +SPT3, (ii) +spt3Δ, (iii) +shuffling], #2 [(i) +spt3Δ, (ii) +SPT3, (iii) +shuffling], and # 3 [(i) +spt3Δ, (ii) +shuffling, (iii) +SPT3]. The two TAF1 and three taf1 strains with the same genetic backgrounds are shaded for clarification. (B) RT-qPCR analyses to measure PGK1 (top panel) or VTC1 (bottom panel) mRNA levels in the ten strains carrying the VTC1 reporter driven by the PGK1 promoter in which the TATA box was intact (lanes 1, 2, 5, 6, and 9) or substituted with the GAGA sequence (lanes 3, 4, 7, 8, and 10), as indicated below the bottom panel. Lane 1–4 strains were generated by protocol #1, lane 5–8 strains were generated by protocol #2, and lane 9–10 strains were generated by protocol #3, as indicated in A. The strain used are YTK19325 (lane 1), YTK19405 (lane 2), YTK19327 (lane 3), YTK19406 (lane 4), YTK19317 (lane 5), YTK19401 (lane 6), YTK19319 (lane 7), YTK19402 (lane 8), YTK19489 (lane 9), and YTK19492 (lane 10). Cultivation and data presentation were conducted as described in Fig 1. (TIF) [file pone.0281233.s001.tif]

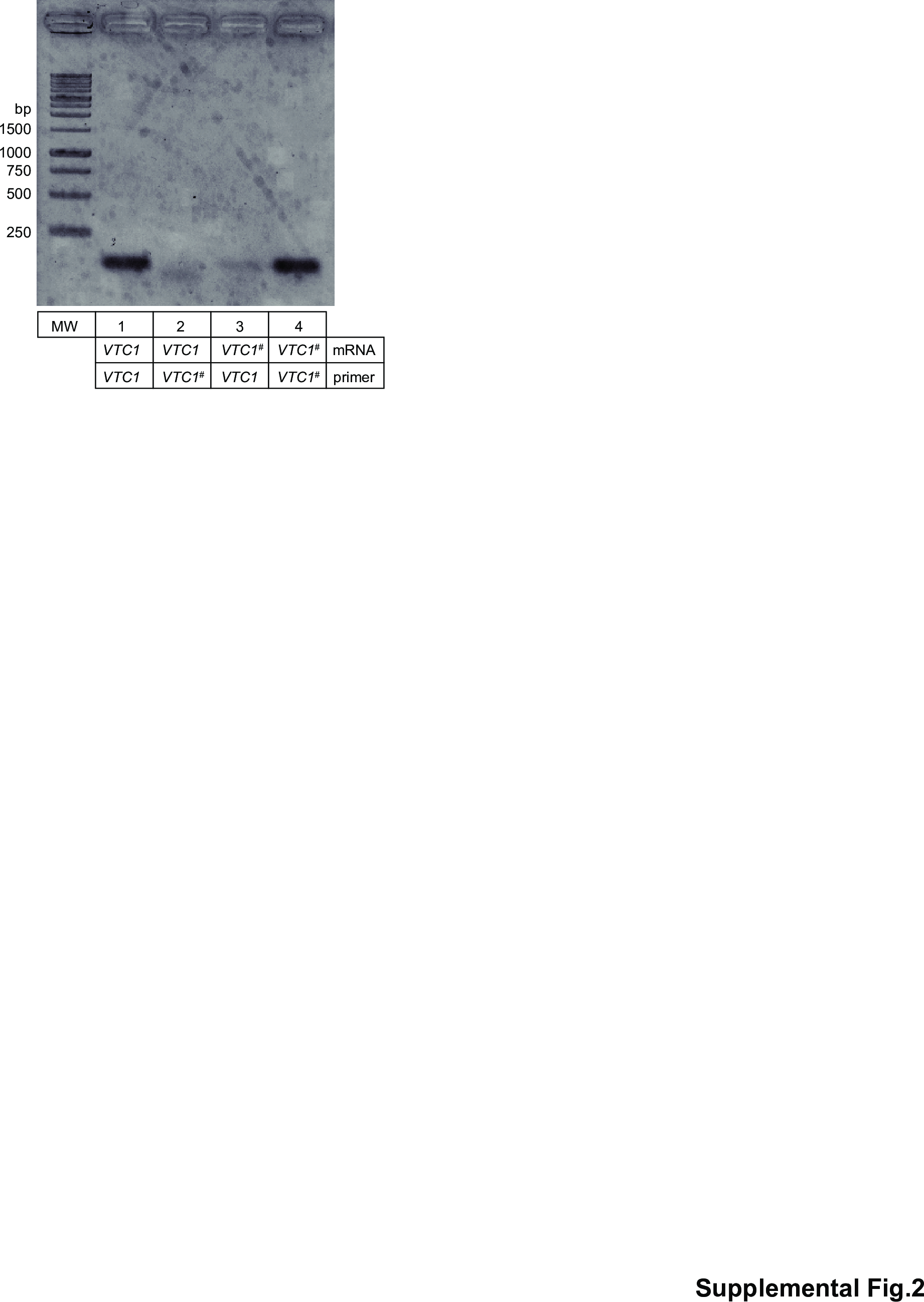

Supplement: S2 Fig — PCR was conducted using cDNA derived from YTK19317 (lanes 1, 2) or YTK19663 (lanes 3, 4) as a template, and primer pairs were VTC1-specific TK13936-TK13937 (lanes 1, 3) or VTC1#-specific TK14458-TK13937 (lanes 2, 4). These cDNA and primer pairs are the same as those used in lanes 1 and 7 of Fig 2B. PCR products were resolved by agarose gel electrophoresis and subsequently visualized by ethidium bromide staining, together with a size marker (MW, ExcelBand 1kb DNA ladder, SMOBIO). (TIF) [file pone.0281233.s002.tif]

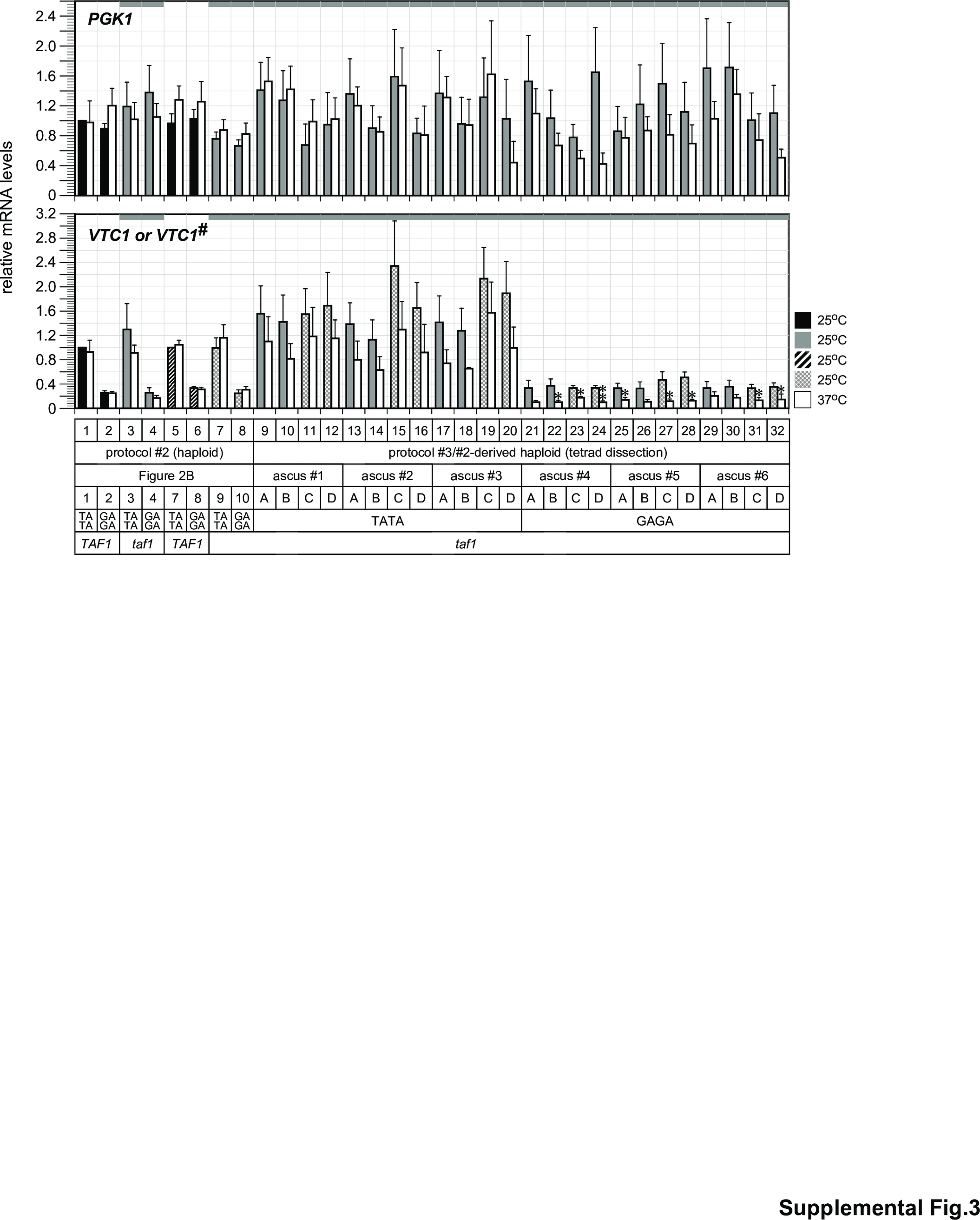

Supplement: S3 Fig — RT-qPCR analysis to measure mRNA levels of PGK1 (top panel) or VTC1/VTC1# (bottom panel) in the 32 haploid strains carrying VTC1 (lanes 1–4, 9–10, 13–14, 17–18, 21–22, 25–26, and 29–30) or the VTC1# reporter (lanes 5–8, 11–12, 15–16, 19–20, 23–24, 27–28, and 31–32) driven by the PGK1 promoter in which the TATA box was intact (lanes 1, 3, 5, 7, and 9–20) or substituted with the GAGA sequence (lanes 2, 4, 6, 8, and 21–32), as indicated below the bottom panel. The strains used in lanes 1–8 were the same as those used in lanes 1, 2, 3, 4, 7, 8, 9, and 10 of Fig 2B, respectively, as indicated. The other strains used in lanes 9–20 and 21–32 were haploid progenitors obtained by tetrad dissection of the mother #3/#2 diploid strains used in lanes 5/9 and 6/10 of Fig 2C, respectively, as indicated. The strains used are YTK19317 (lane 1), YTK19319 (lane 2), YTK19401 (lane 3), YTK19402 (lane 4), YTK19663 (lane 5), YTK19665 (lane 6), YTK19664 (lane 7), YTK19666 (lane 8), YTK19803 (lane 9), YTK19805 (lane 10), YTK19804 (lane 11), YTK19806 (lane 12), YTK19807 (lane 13), YTK19808 (lane 14), YTK19809 (lane 15), YTK19810 (lane 16), YTK19813 (lane 17), YTK19814 (lane 18), YTK19811 (lane 19), YTK19812 (lane 20), YTK19817 (lane 21), YTK19818 (lane 22), YTK19815 (lane 23), YTK19816 (lane 24), YTK19819 (lane 25), YTK19821 (lane 26), YTK19820 (lane 27), YTK19822 (lane 28), YTK19823 (lane 29), YTK19825 (lane 30), YTK19824 (lane 31), and YTK19826 (lane 32). Cultivation and data presentation were conducted as described in Fig 1A. (TIF) [file pone.0281233.s003.tif]

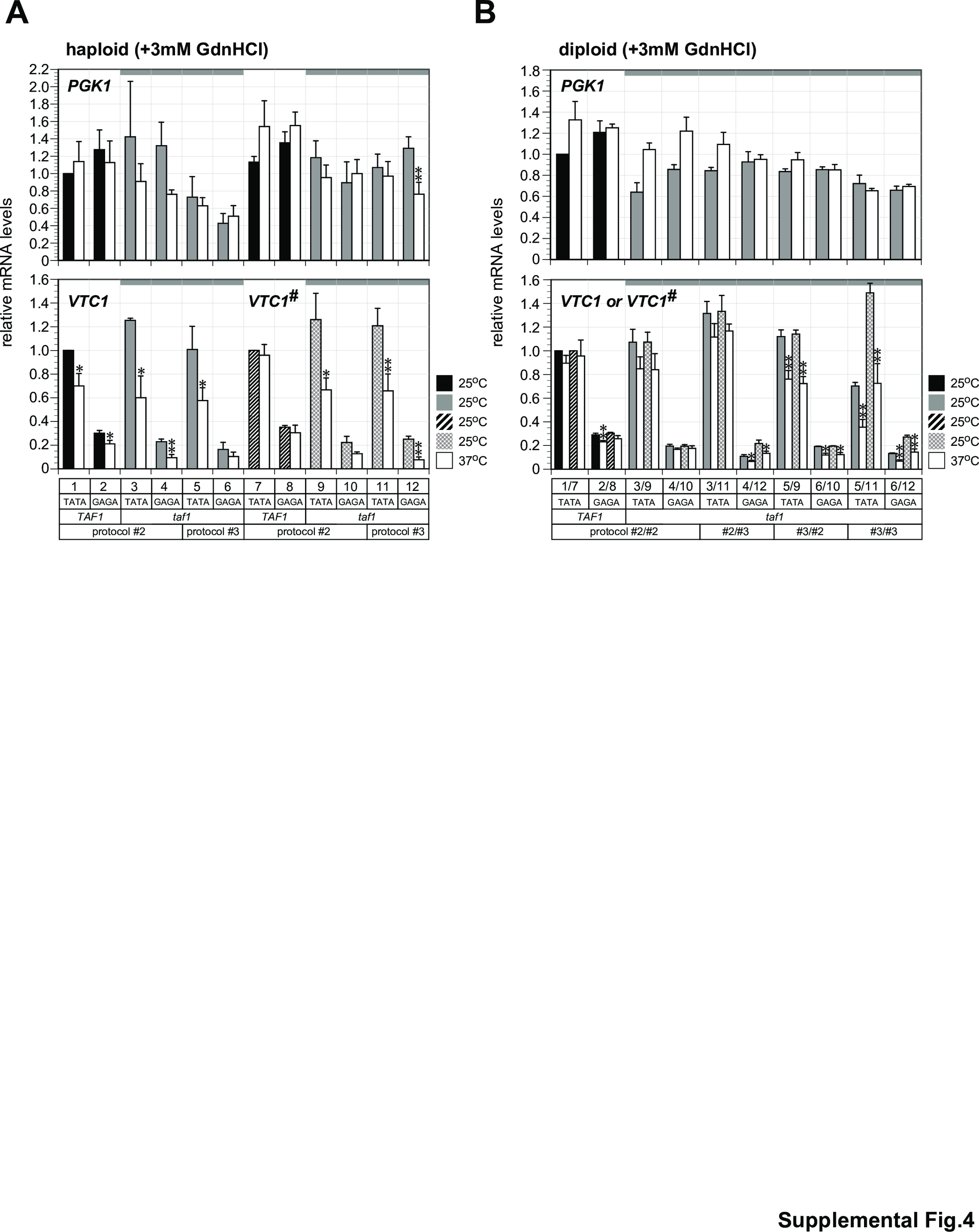

Supplement: S4 Fig — (A) RT-qPCR analysis to measure mRNA levels of PGK1 (top panel) or VTC1/VTC1# (bottom panel) in the twelve haploid strains carrying VTC1 (lanes 1–6) or the VTC1# reporter (lanes 7–12) driven by the PGK1 promoter in which the TATA was intact (odd-numbered lanes) or substituted with the GAGA sequence (even-numbered lanes), as indicated below the bottom panel. These strains are the same as those used in Fig 2B. Cultivation and data presentation were conducted as described in Fig 1A, except that the media used here contained 3 mM Gdn-HCl. (B) RT-qPCR analysis to measure mRNA levels of PGK1 (top panel) or VTC1/VTC1# (bottom panel) in the ten diploid strains carrying both of the VTC1 and VTC1# reporters driven by the PGK1 promoter in which the TATA box was intact (lanes 1/7, 3/9, 3/11, 5/9, and 5/11) or substituted with the GAGA sequence (lanes 2/8, 4/10, 4/12, 6/10, and 6/12), as indicated below the bottom panel. The strains are the same as those used in Fig 2C. Cultivation and data presentation were conducted as described in Fig 1A, except that the media also contained 3 mM Gdn-HCl. (TIF) [file pone.0281233.s004.tif]

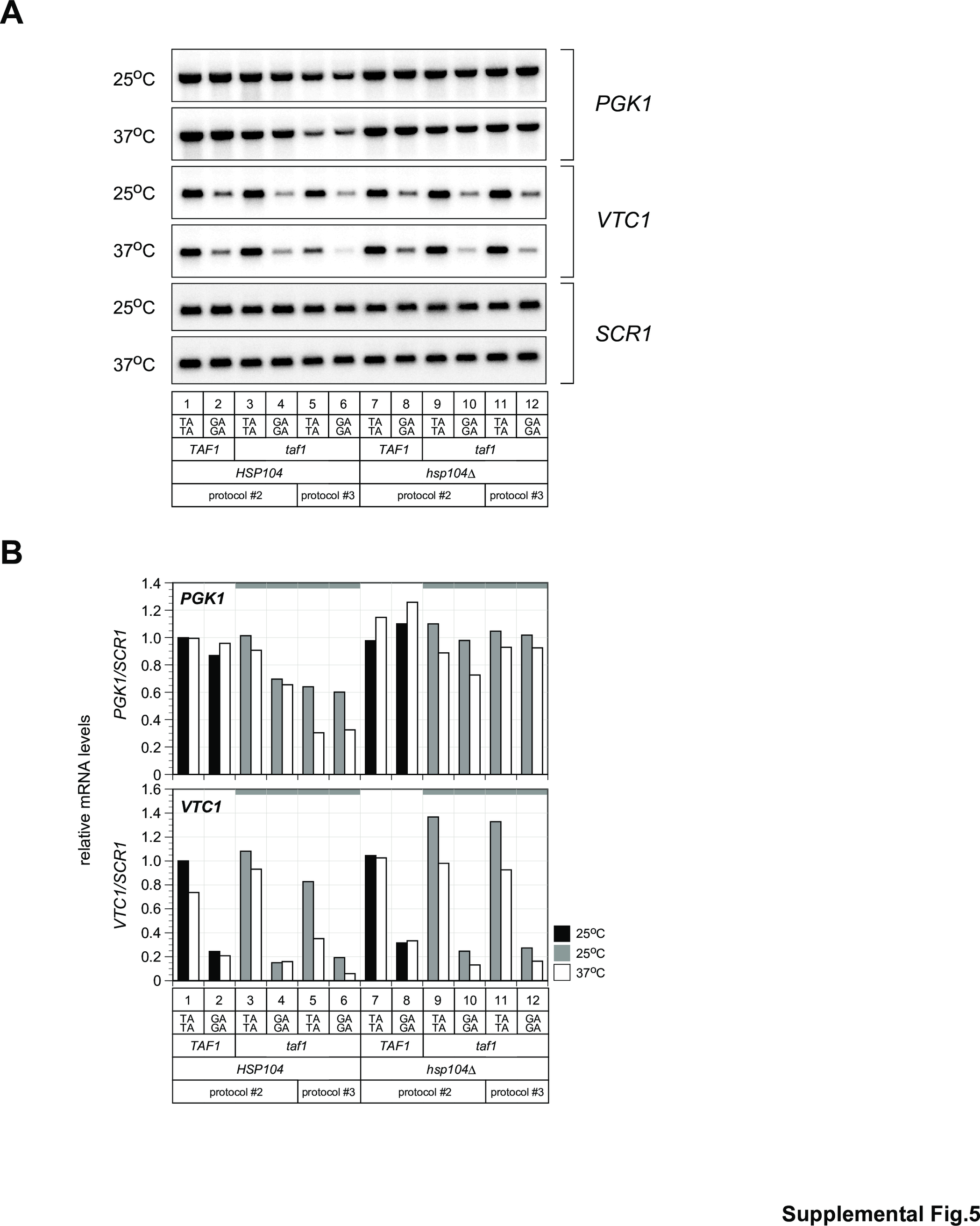

Supplement: S5 Fig — (A) Northern blot analysis of VTC1 (middle panels), PGK1 (top panels), or SCR1 (control; bottom panels) RNA levels in the twelve strains used in Figs 2 (lanes 1–6) and 3 (lanes 7–12). The strains used are YTK19317 (lane 1), YTK19319 (lane 2), YTK19401 (lane 3), YTK19402 (lane 4), YTK19489 (lane 5), YTK19492 (lane 6), YTK20037 (lane 7), YTK20039 (lane 8), YTK20038 (lane 9), YTK20040 (lane 10), YTK20049 (lane 11), and YTK20050 (lane 12). Cultivation was performed as described in Fig 1A. (B) Raw expression data in A were quantified and normalized to SCR1. Values for each transcript derived from PGK1 or VTC1 are summarized in the upper or lower panel, respectively. In each panel, data are presented relative to the value obtained for the strain indicated on the left. (TIF) [file pone.0281233.s005.tif]

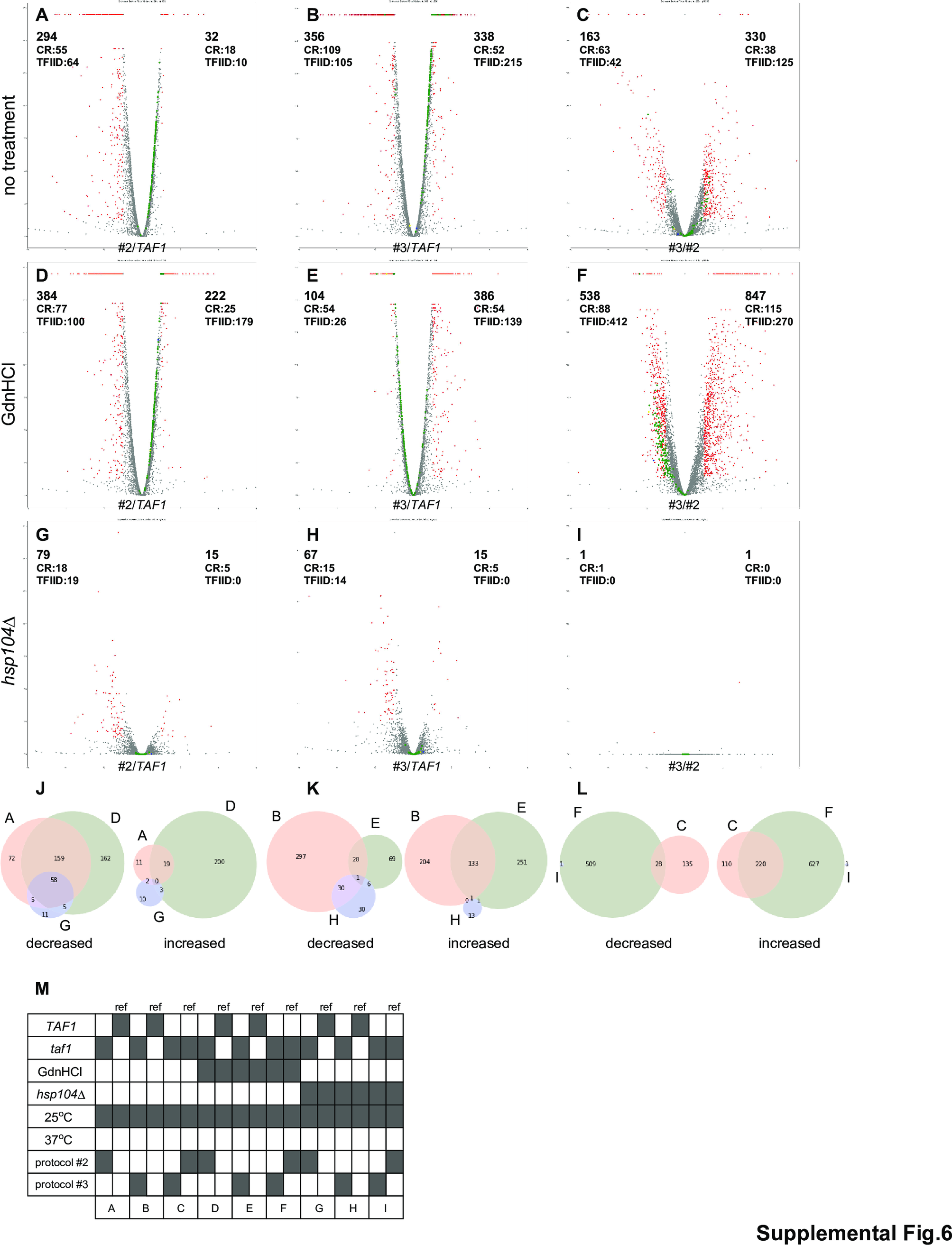

Supplement: S6 Fig — TPM data of RNA-seq analyses obtained from two biological replicates of six strains carrying or not carrying taf1 or hsp104Δ mutations were averaged and subjected to volcano plot analyses as shown in Fig 4. Note that the data analyzed in this figure are derived from strains cultured at 25°C, while those analyzed in Fig 4 of the main text are derived from the strains cultured at 37°C. The analyzed data (A–L) and cultivation/comparison details (M) are represented as described in Fig 4. (TIF) [file pone.0281233.s006.tif]

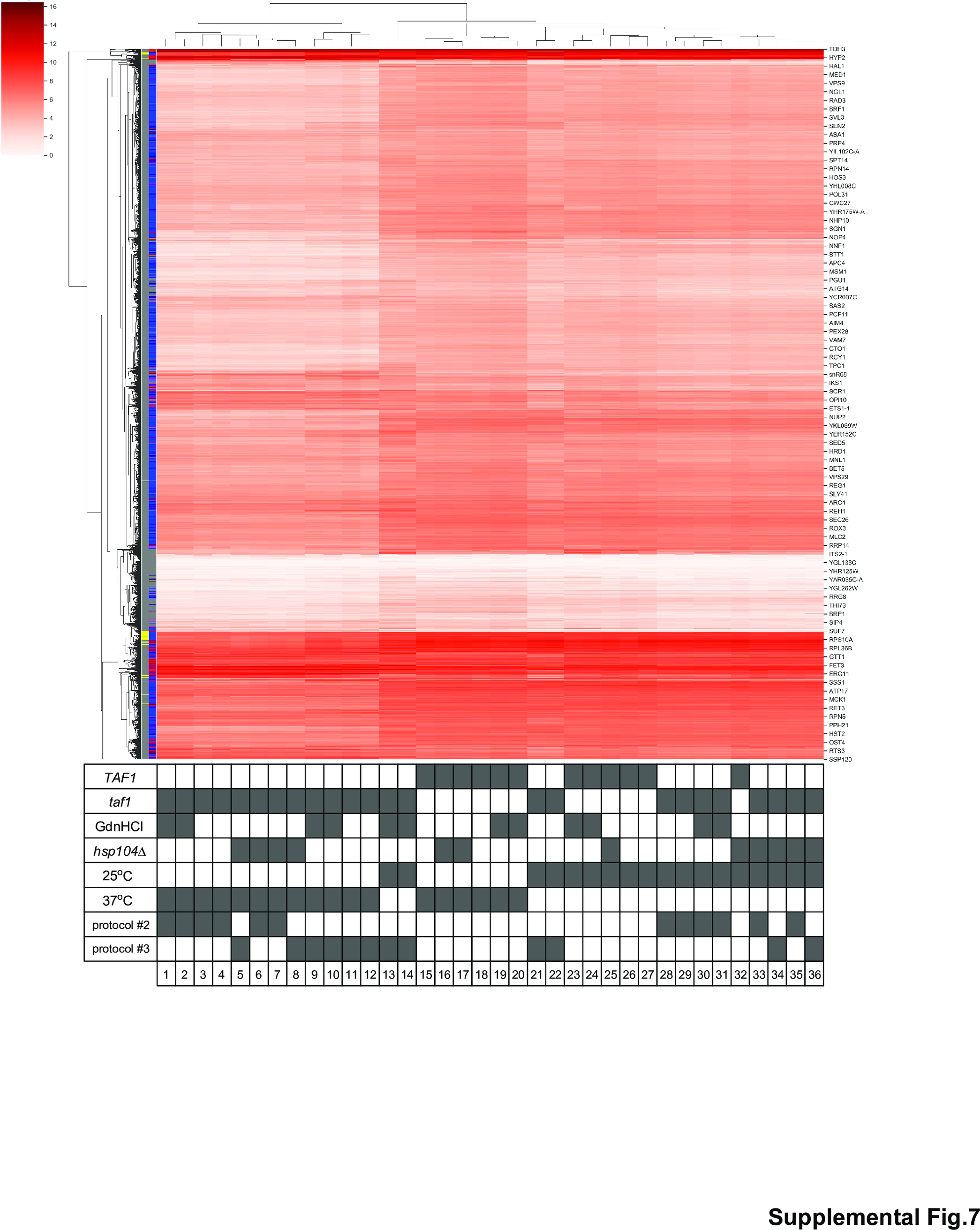

Supplement: S7 Fig — Duplicate TPM data from six strains carrying or not carrying taf1 or hsp104Δ mutations after cultivation under the conditions indicated in the bottom panel were subjected to hierarchical clustering analysis and visualized as a heat map. The same data were analyzed by volcano plot in Fig 4 and S6 Fig. Ribosomal protein genes (yellow), coactivator-redundant (CR) genes (red), and TFIID-dependent genes (blue) are indicated on the left side of the heatmap. Note that the gene list indicated on the right side of the heatmap is incomplete due to space limitations. The strains used are YTK19317 (columns #15, 18–20, 23–24, and 26–27), YTK19401 (columns #1–4 and 28–31), YTK19489 (columns #9–14 and 21–22), YTK20037 (columns #16–17, 25, and 32), YTK20038 (column #6–7, 33, and 35), and YTK20049 (columns #5, 8, 34, and 36). Cultivation was performed as described in Fig 1A and S4 Fig. (TIF) [file pone.0281233.s007.tif]

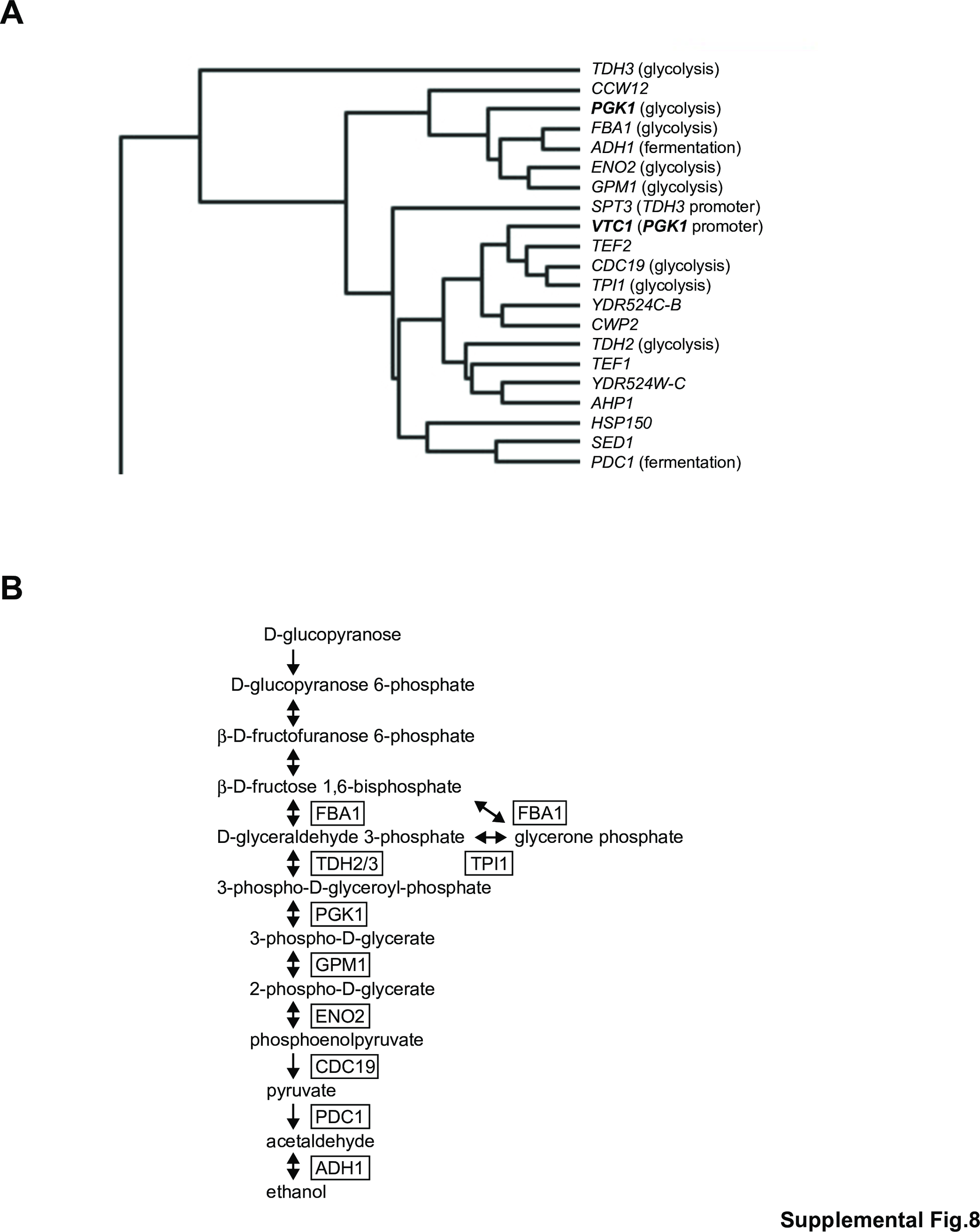

Supplement: S8 Fig — The cluster comprising the 21 genes and located at the top of the heatmap in S7 Fig is enlarged for detail. Note that the SPT3 and VTC1 genes are driven by the TDH3 and PGK1 promoters, respectively, in the strains subjected to RNA-seq analyses. (A) The metabolic pathways from glucose to ethanol (glycolysis and ethanol fermentation) are summarized with the names of gene products (open rectangles) catalyzing the corresponding reactions, if they are present in the cluster shown in A. (TIF) [file pone.0281233.s008.tif]
